# 4-Methoxydalbergione Inhibits Bladder Cancer Cell Growth *via* Inducing Autophagy and Inhibiting Akt/ERK Signaling Pathway

**DOI:** 10.3389/fmolb.2021.789658

**Published:** 2022-02-16

**Authors:** Haifang Du, Ting Tao, Simeng Xu, Changqiong Xu, Shan Li, Qiongli Su, Jing Yan, Bo Liu, Ran Li

**Affiliations:** ^1^ The Second Clinical Medical College, Guangdong Provincial Key Laboratory of Clinical Research on Traditional Chinese Medicine Syndrome, Guangzhou University of Chinese Medicine, Guangzhou, China; ^2^ Guangzhou Key Laboratory of Chirality Research on Active Components of Traditional Chinese Medicine, Guangzhou, China; ^3^ Scientific Research Institute, Yueyang Maternal-Child Medicine Health Hospital, Yueyang, China; ^4^ Key Laboratory of Study and Discovery of Small Targeted Molecules of Hunan Province, Key Laboratory of Protein Chemistry and Developmental Biology of Fish of Ministry of Education, Department of Pharmacy, School of Medicine, Hunan Normal University, Changsha, China; ^5^ Department of Pharmacy, Zhuzhou Central Hospital, Zhuzhou, China

**Keywords:** 4MOD, bladder cancer cell, apoptosis, autophagy, Akt/Erk signaling

## Abstract

Bladder cancer (BC) ranks the fourth in incidence in cancers of men and is a common malignant tumor in women. 4-Methoxydalbergione (4MOD), which is purified from Dalbergia sissoo Roxb, has been shown to have anticancer capacity for osteosarcoma and astroglioma. The role of 4MOD in bladder cancer has not been investigated. This study aims to evaluate the anticancer effect of 4MOD in BC cells and its possible mechanisms. The two human bladder cancer cell lines J82 and UMUC3 were used to evaluate the proliferation inhibitory effect of 4MOD by CCK8 and clonogenic assays. The migratory and invasive ability of tumor cells was examined by scratch test and transwell assay. Apoptosis was detected by flow cytometry and TUNEL assays. The autophagy-related molecules including Beclin-1 and LC3 were examined by Western blotting analysis. Furthermore, the RT-PCR was used to detect the mRNA expression of LC3. 4MOD repressed cell proliferation, migration, invasion and induced cell apoptosis in a concentration-dependent manner. The IC_50_ values of J82 and UMUC3 were 8.17 and 14.50 μM respectively. The mRNA and protein expression ratio of light chain 3-II (LC3-II)/LC3-I and the protein expression of Beclin-1 were increased when the BC cells were treated with 4MOD. The treatment of 4MOD attenuated the phosphorylation of Akt and ERK in the BC cells. We revealed that the 4MOD inhibits BC cells growth by inducing autophagy and inhibiting Akt/ERK signaling pathway. Our study provides new insights into the mechanism by which 4MOD weakens the proliferation of BC cells. This study demonstrates that 4MOD provided a lead compound for the development of novel compound with potent anticancer effect on BC cells.

## Introduction

Bladder cancer is one of the most common malignancies in the urinary system worldwide (Sanli et al., 2017). There are more than 430,000 confirmed cases of bladder cancer in the world every year (Siegel et al., 2018). Radical cystectomy (RC) combined with lymph node dissection (LND) is recommended as the standard treatment for muscle-invasive bladder cancer (MIBC). Patients with low and intermediate risk of non-muscle-invasive bladder cancer (NMIBC) have a 5-years recurrence-free survival rate of 43 and 33%, respectively (Kohada et al., 2021). However, 50–70% of cases are myometrial invasive bladder cancer (MIBC) and the 5-years overall survival (OS) is only 4.8% due to the devastating metastasis (Bokarica et al., 2017). The incidence and mortality remained low before 45 and 55 years old and reached the peak in the age group of 80 years (Li et al., 2021). Furthermore, radio- resistance and drug-resistance often occur. Hence, development of more practical drug candidates and therapeutic strategy is urgently needed.

4-Methoxydalbergione(4MOD), was isolated and purified from the heartwood of *Dalbergia sissoo* Roxb. Recent research has shown the anticancer capacity of 4MOD for osteosarcoma (Park et al., 2016) and astroglioma (Li et al., 2020). In addition, 4MOD showed cytoprotective effects on hippocampal BV2 microglia cells in mice and primary microglia in rats (Bokarica et al., 2017). Therefore, as a natural flavonoid, 4MOD has a good prospect of development into an anticancer medication. However, the anticancer effect of 4MOD in bladder cancer cells remains unclear.

Autophagy is a membrane transport and intracellular degradation system which captures abnormal intracellular dysfunctional organelles and deliver them to lysosomes. Autophagy affects cellular homeostasis, aging and diverse diseases (Zhao et al., 2021). Autophagy is a double-edged sword in the development of cancers due to the dual effects of inhibiting and promoting tumorigenesis. Presently, autophagy regulation is used in anti-tumor treatment in different cancer types (Zhao et al., 2021). More than 80 clinical trials based on autophagy are in progress. (https://clinicaltrials.gov/; accessed on april 12, 2021). In recent years, the role of autophagy in bladder cancer has been studied. Riccardo Vanzo group found that autophagy is enhanced in both early and advanced stages of human urinary bladder tumorigenesis (Vanzo et al., 2020). It was shown that treatment with the autophagy inhibitor bafilomycin A1 or knocking down the autophagy-related protein ATG7 or 12 can induce a large number of cell apoptosis and leading to the death of bladder cancer cells (Lin et al., 2016). Furthermore, the knockdown of ATG7 dramatically inhibits human BC cell invasion (Zhu et al., 2019).

Recently, the development of therapeutic drugs for bladder cancer targeting the autophagic pathway has become a hot spot in the field of bladder cancer research. It has been reported that the autophagy-modulating drug arzanol can induce the accumulation of lapidated LC3 and sensitize RT-112 bladder cancer cells to cisplatin (CDDP) treatment (Deitersen et al., 2021). ChlA-F, a novel conformation-derivative of Cheliensisin A, induces autophagy-dependent anticancer effects through specifically promoting SESN2 expression (Hua et al., 2018). SESN2, as a new p53 targeted gene, is involved in the induction of autophagy (Maiuri et al., 2009), and overexpression of SESN2 mediates the anti-cancer activity of Isorhapontigenin in bladder cancer cells (Liang et al., 2016). A large number of studies have shown that autophagy is related to drug resistance in the treatment of bladder cancer with gemcitabine, cisplatin, and epirubicin (Huang et al., 2017; Yin et al., 2017).

Various signaling pathways play important roles in cell proliferation, apoptosis and autophagy on cancer development (Li D. et al., 2018). Akt/ERK signaling pathway stimulates cell proliferation at various stages of the cell cycle. Inhibition of Akt/ERK signaling pathway is a good anti-tumor strategy (Huang et al., 2017; Yin et al., 2017). However, the effect of 4MOD on autophagy and Akt/ERK signaling pathway in the development of bladder cancer cell is elusive.

In this study, we aim to examination the anticancer effects of 4MOD and the underlying mechanism on the development of BC cells. Our results confirm that 4MOD is a new and promising compound, which inhibits the growth and induces apoptosis of BC cells by preventing Akt/ERK signaling pathway from activating autophagy.

## Materials and Method

### Cell Culture

Human BC cell lines J82 and UMUC3 (provided by Dr. P Guo) were cultured in Dulbecco modified Eagle medium supplemented (Hyclone, Logan, UT) with 10% of BI serum (FBS) (Hyclone, Logan, UT) and 1% of penicillin-streptomycin at 37°C, in humidified air containing 5% of CO_2_.

### Reagents

4MOD (98% purity, MW = 254.28) was isolated and purified from *Dalbergia sissoo* Roxb. in our preliminary study (Ran et al., 2019). and it prepared as a stock solution of 50 mM in dimethyl sulfoxide (DMSO). SC79, and CQ (Selleck-Biotool, Shanghai, China) were prepared as a stock solution of 50 mM in PBS. They were diluted in dimethyl sulfoxide in a range of concentrations. Antibodies against Akt, ERK, LC3, GAPDH and *β*-actin were purchased from Cell Signaling Technology (Cell Signaling, Beverly, MA). An Apoptosis Detection kit (FITC Annexin V) was purchased from Beyotime (CS, Hunan, China). The TUNEL Assay Kit was obtained from Elabscience. MA.

### Cell Viability

Cell proliferation was determined by using a Cell Counting Kit-8 assay (CCK-8, Meilunbio, Dalian, China). Cells were seeded into a 96-well plate (6 × 10^3^ cells/well) with 100 μL of culture medium. After 24 h, cells were treated with different drugs and incubated for 72 h. 10 μL CCK-8 solution was added to each well and cells were incubated at 37°C for 2 h. Light absorbance was measured at 450 nm using a Dynatech MR5000 plate reader.

### Colony Formation Assays

A total of 6×10^3^ cells were seeded into 24-well dishes in 0.5 ml of medium for 24 h. Cells were further treated with different drugs, and then maintained for 6–8 days in a CO_2_ incubator. Finally, the cells were fixed with 10% formaldehyde solution and then stained with 0.1% crystal violet, each experiment was performed in triplicate. The absorbance was measured using a microplate reader at 550-nm wavelength.

### Transwell Assays

Transwell assay was used to analyze the impacts of different concentrations of 4MOD (0 μM, 5.0 μM, 10 μM) on cell migration and invasion of BC cells by applying a Transwell plate (Corning, United States) according to the manufacturer’s guidance. Briefly, the upper chambers were plated with around 1×10^5^ cells. Then the cells were fixed in paraformaldehyde (4%) and dyed using crystal violet. Invaded and migrated cells were recorded and calculated.

### Measurement of Cell Migration

A total of 5×10^3^ cells were seeded onto a 12-well plate and allowed to reach full confluence. The monolayer was wounded using a cocktail stick. Cells were incubated with serum-free MEM medium, for the time period as stated. Digital images were taken at times 0, 24 and 48 h. The mean area was calculated using ImageJ software. The experiments were repeated three times.

### TUNEL Assays

Apoptosis was analyzed using a TUNEL Apoptosis Detection kit (Elabscience, Institute of Biotechnology) according to the manufacturer’s protocol. The nuclei were counterstained with DAPI for 5 min at room temperature in the dark and the slides were then mounted with anti-fade mounting medium. The levels of apoptosis were estimated as the ratio of the number of TUNEL-positive cells to the total number of DAPI-positive cells using a fluorescence microscope (magnification, ×200; Olympus Corporation).

### Annexin V-FITC/PI Cell Apoptosis Analysis

Apoptosis was detected by flow cytometry *via* the examination of altered plasma membrane phospholipid packing by lipophilic dye Annexin V. Briefly, treated cells were harvested by trypsin, washed twice with PBS, and then resuspended in binding buffer at a concentration of 5.0 × 10^5^ cells/mL according to the manufacturer’s instruction. Thereafter, 5 μL of Annexin V-FITC and 10 μL of propidium iodide were added into 100 μL of cell suspension and incubated for 30 min at room temperature in the dark. After adding 300 μL of binding buffer, labeled cells were counted by flow cytometry within 30 min. All early apoptotic cells (Annexin V-positive and propidium iodide-negative), necrotic/late apoptotic cells (double positive), as well as living cells (double negative) were detected by flow cytometry on a FACS Calibur flow cytometer.

### qRT-PCR Analysis

Total RNA was isolated using a Total RNA Extraction kit (GeneCopoeia™, Guangzhou, China). Reverse transcription into cDNA was performed using a SureScript™ First-Stand cDNA Synthesis Kit according to the User Manual (GeneCopoeia™, Guangzhou, China) and the cDNA was amplified using a SYBR^®^ Green qPCR Mix 2.0 (GeneCopoeia™, Guangzhou, China). The 2^−ΔΔCt^ method was used to perform analysis with 18 s RNA as the reference. The primers of LC3 were purchased from GeneCopoeia™. Data analysis was performed with Applied Biosystems Quant Studio 3 Real-time PCR System (Thermo Fisher Scientific, United States).

### Western Blot Analysis

Western blot assessment was performed using regular procedures. Primary antibody was added in bovine serum albumin (BSA) and allowed to incubate overnight at 4°C, washed with TBST for six times (10 min per time). The secondary antibodies (CST, United States) were used for hatching the membranes 1 h at room temperature. The Pierce Super Signal chemiluminescent substrate (Rockford, IL, United States) was added followed by the visualization with a Fluorescentand Chemiluminescence Gel Imaging System (Jiapeng, ShangHai, China). Band intensity data were quantified using ImageJ.

### Statistical Analysis

Data were expressed as mean ± SD, and the statistical analysis was conducted using GraphPad prism 9. The unpaired Student’s *t*-test was used to compare two groups, and the one-way *ANOVA* was used to compare among multiple groups. *p < 0.05, **p < 0.01 was considered as statistically significant.

## Results

### 4MOD Inhibits BC Cell Proliferation and Colony Formation

**FIGURE 1:**
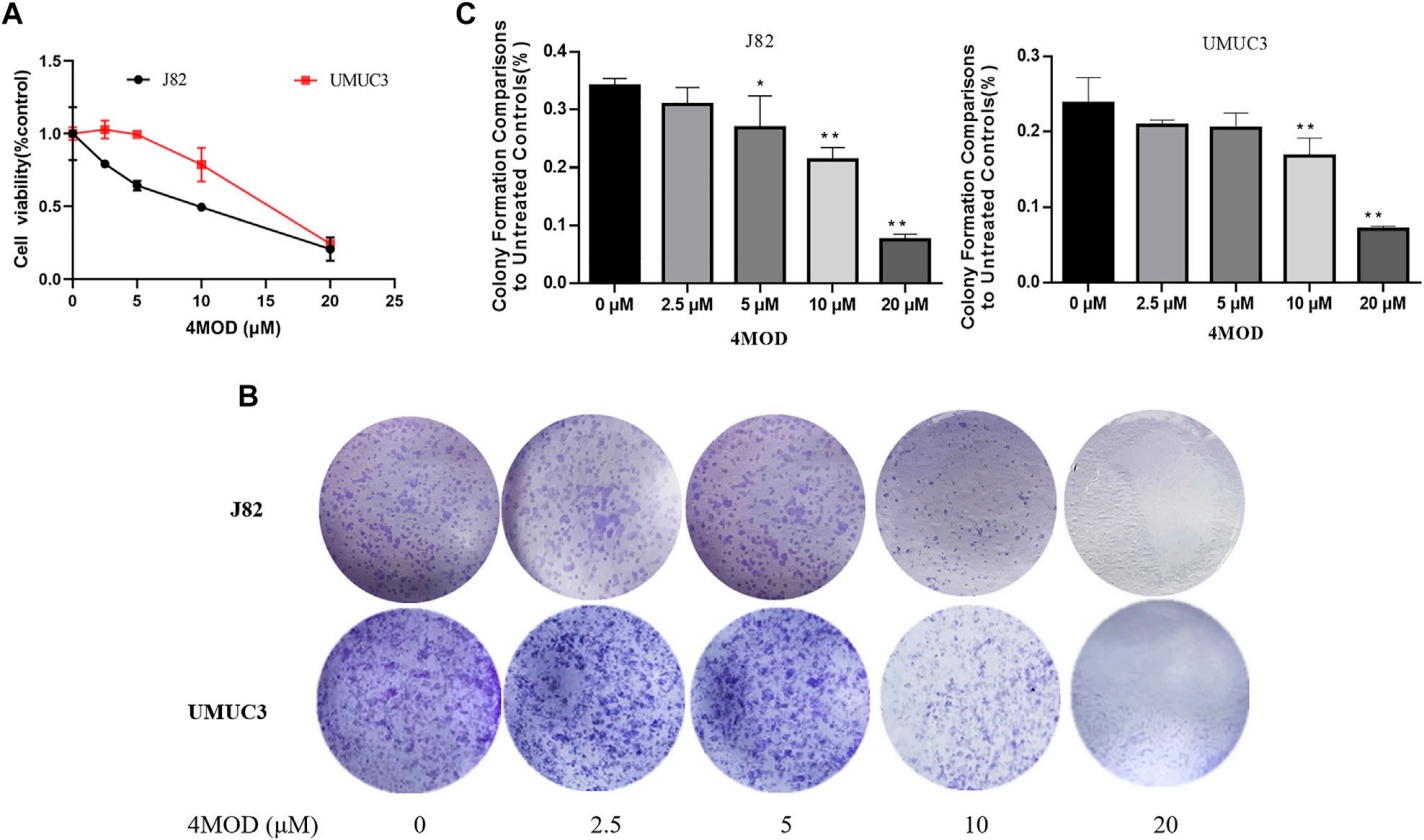
4MOD inhibits the proliferation of BC cells **(A)** CCK8 assay on the human BC cell lines UMUC3 and J82 treated with 4MOD at concentrations ranging from 0 to 20 μM or DMSO for 72 h. **(B)** The clonogenic assay assessed after 7-days of 4MOD treatment at various concentrations (0–20 μM) **(C)** Bar chart shows quantitative data of average of 3 independent experiments (*p < 0.05, **p < 0.01 compared with control). Quantitative data determined by solubilization of crystal violet and spectrophotometric reading at OD 550 nm. Results are presented as mean ± SD of 3 independent experiments (Comparison was drawn by the *t*-test (two-tailed). Data represent means ± SD; *p < 0.05, **<0.01).

We used Cell Counting Kit-8 analysis to verify whether 4MOD affects the proliferation of BC cells *in vitro*. We exposed J82 and UMCU3 cells to various concentrations of 4MOD (0 μM, 2.5 μM, 5.0 μM, 10 μM, 20 μM). It was found that 4MOD inhibited cell proliferation in a dose-dependent manner (Figure 1A; Table 1). The IC_50_ values of 4MOD induced inhibition at various concentrations were 8.17 μM, 14.5 μM on bladder cancer cells, respectively (Figure 1A; Table 1). Similarly, the colony formation was decreased by treatment with 0–20 μM 4MOD (Figures 1B,C). The J82 is the more sensitive cell line which is consistent with the results of CCK8 assay.

**TABLE 1:** Inhibitory concentration 50% (IC_50_) of 4MOD.

	J82	UMUC3
IC_50_	8.17 ± 1.91 μM	14.50 ± 0.92 μM

### 4MOD Represses BC Cell Invasion and Migration

**FIGURE 2:**
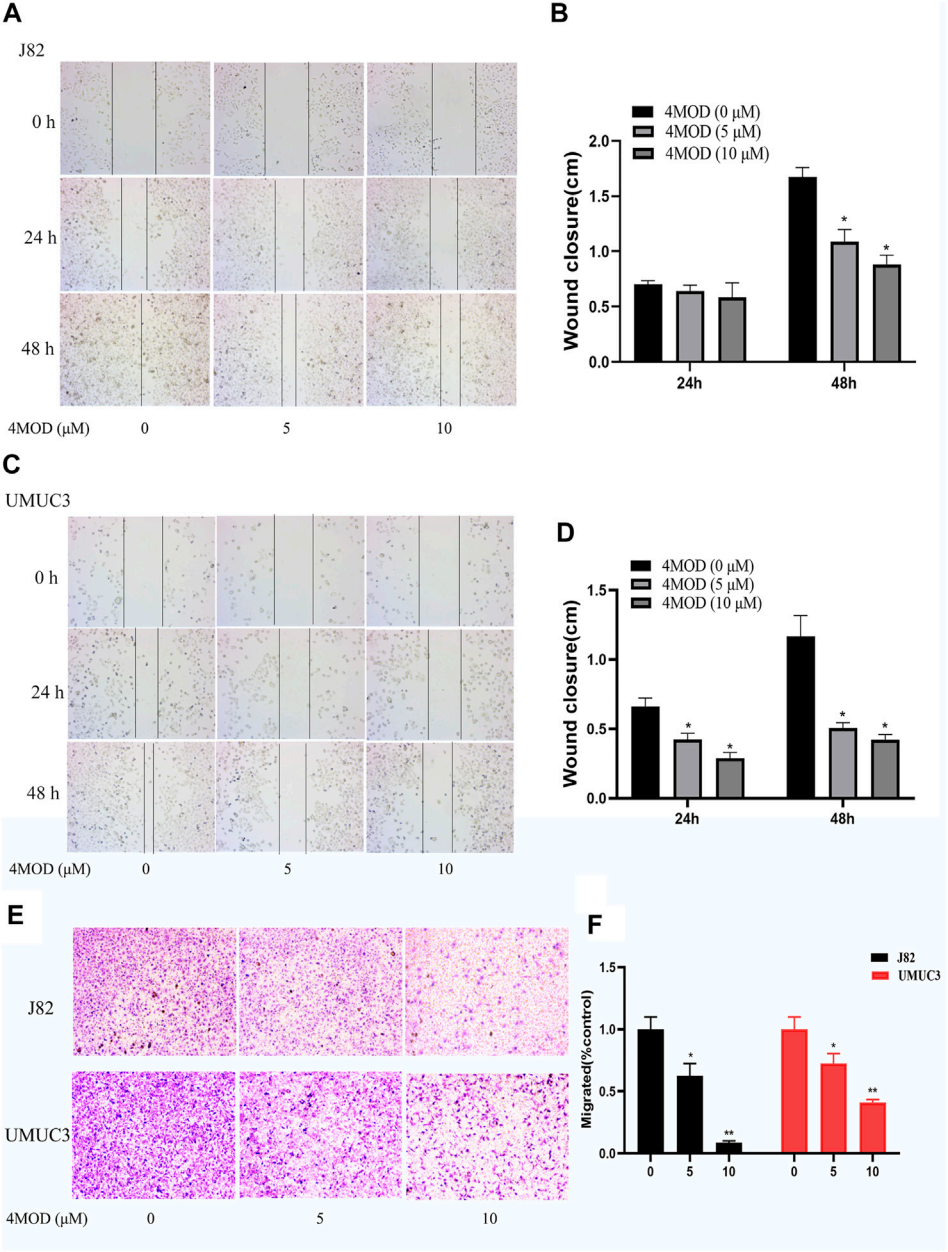
4MOD represses invasion and migration of BC cells **(A–D)** Transwell assay on the human BC cell lines UMUC3 and J82 treated with 4MOD (0 μM, 5.0 and 10 μM)) for 48 h **(E–F)** The migration was measured by wound healing assays in the different group of cells; Data are presented as mean ± SD of 3 independent wound healing assays experiments (*p < 0.05, **p < 0.01 compared with control).

Cell scratch assay and Transwell assay were used to analyze the effect of 4MOD on the migration and invasion of BC cells. As shown in Figure 2, 4MOD-treated cells were remarkably enhanced the wound healing proportion than that of control in J82 and UMUC3 cells at 24 and 48 h (Figures 2A–D), Similarly, Transwell assays results showed that the invasion was decreased in the cells treated with 0–20 μM 4MOD (Figures 2E,F), suggesting that 4MOD can decrease the migration and invasion of BC cells *in vitro*.

### 4MOD Promotes Apoptosis of BC Cells

**FIGURE 3:**
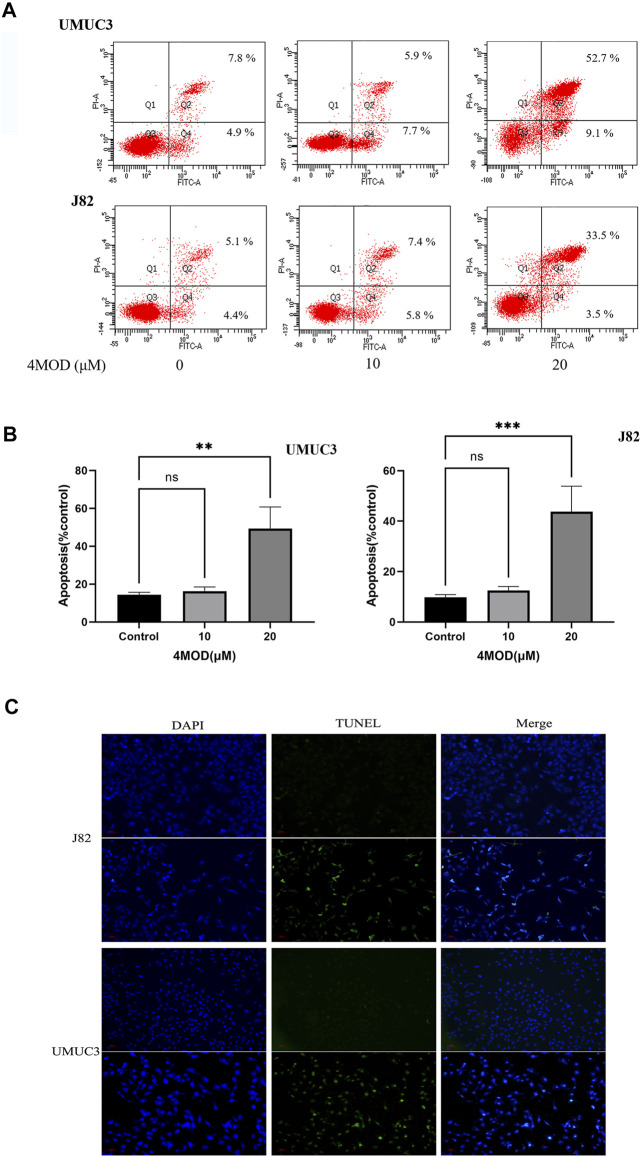
4MOD promotes apoptosis of BC cells. The cell apoptosis was measured by flow cytometry analysis **(A-B)** and TUNEL assays **(C)** in BC cells of J82 and UMUC3. Data are presented as mean ± SD. Statistic significant differences were indicated: *p < 0.05, **p < 0.01, ***p < 0.001.

As shown in Figure 3, compared to the control group, the cellular apoptosis was remarkably increased in the cells treated with 20.0 µM 4MOD, as demonstrated by both FITC and PI staining (Figures 3A,B). Consistently, as we can see in Figure 3C under a fluorescence microscope, 4MOD-treated cells significantly increased TUNEL-positive staining (green fluorescence) compared with the control group. These data demonstrated that 4MOD is able to induce apoptosis of BC cells.

### 4MOD Induces Autophagy of BC Cells

**FIGURE 4:**
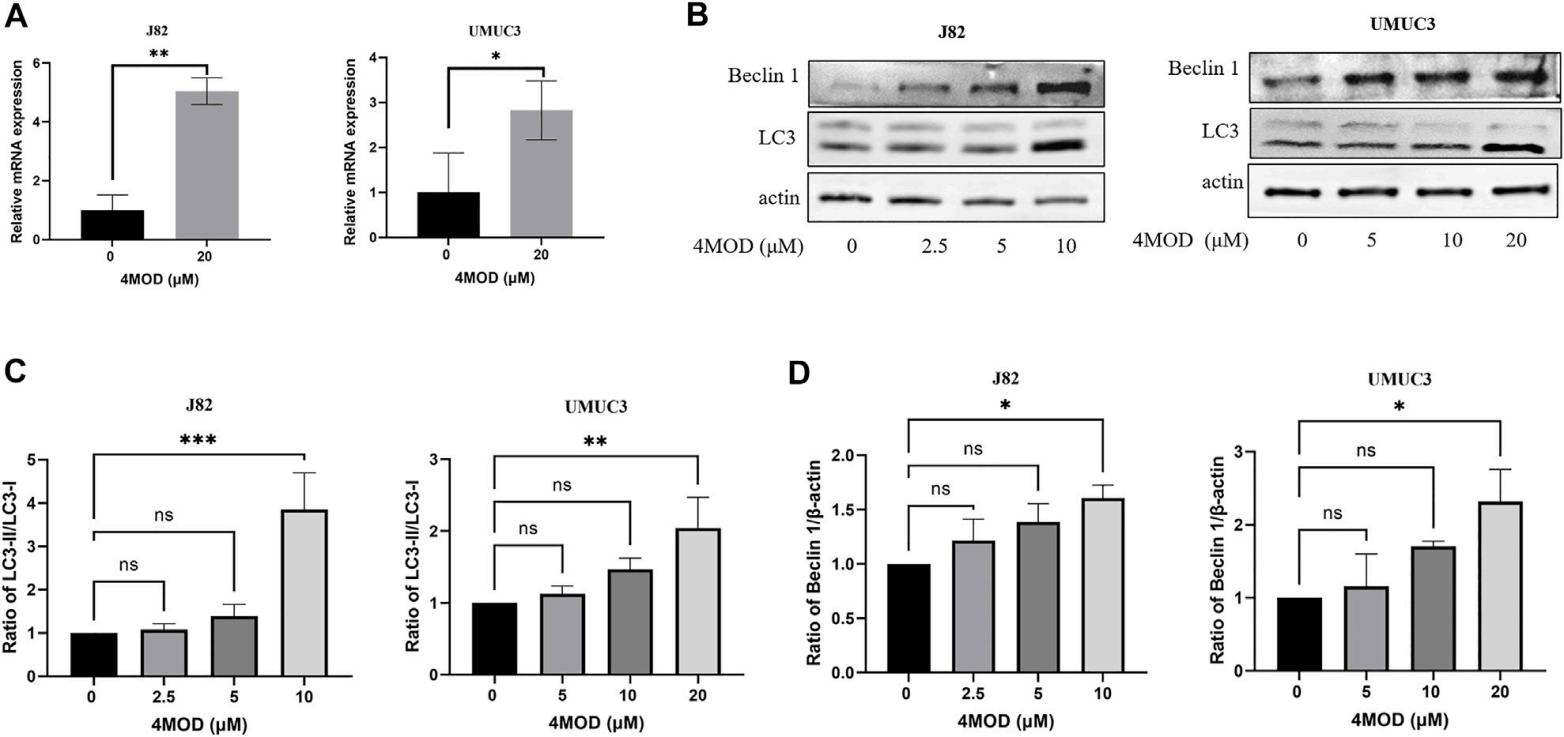
4MOD induces autophagy in BC cells **(A)** The human BC cell lines UMUC3 and J82 were treated with 4MOD (20 μM) for 12 h. The relative mRNA expression of LC3 was measured by RT-PCR **(B)** The UMUC3 and J82 cells were treated with an increasing concentration of 4MOD for 72 h. The expression of LC3-II, LC3-I, Beclin-1, and β-actin was measured by Western blot analysis in the cells **(C–D)** The results of Western blot analysis were quantified by ImageJ software. Data are presented as mean ± SD. Statistic significant differences were indicated: *p < 0.05, **p < 0.01.

Abnormal autophagy is closely related to the occurrence and development of bladder cancer (Zhu et al., 2019). Salidroside significantly increased the autophagy of T24 cells through the autophagy/PI3K/Akt and MMP-9 signaling induced bladder cancer cells apoptosis (Li T. et al., 2018). Based on this, we explored whether 4MOD affects the progression of bladder cancer cells through autophagy. As shown in Figure 4A, We found that 4MOD significantly enhanced LC3 mRNA expression. LC3, is an autophagic landmark gene, which participates in the formation of the autophagosome (Giménez-Xavier et al., 2009). At the same time, we further identified that 4MOD induced the expression ratio of LC3 (LC3B-II)/LC3B-I and the protein expression of Beclin-1 in J82 and UMUC3 cells in a dose-dependent manner Figure 4B. The statistical analysis of protein quantifications in Figures 4C,D verified it (*p < 0.05). These results suggested that 4MOD induces autophagy of BC cells.

### Antagonism Action of Combination of 4MOD With CQ on Proliferation and Colony Formation in BC Cells

**FIGURE 5:**
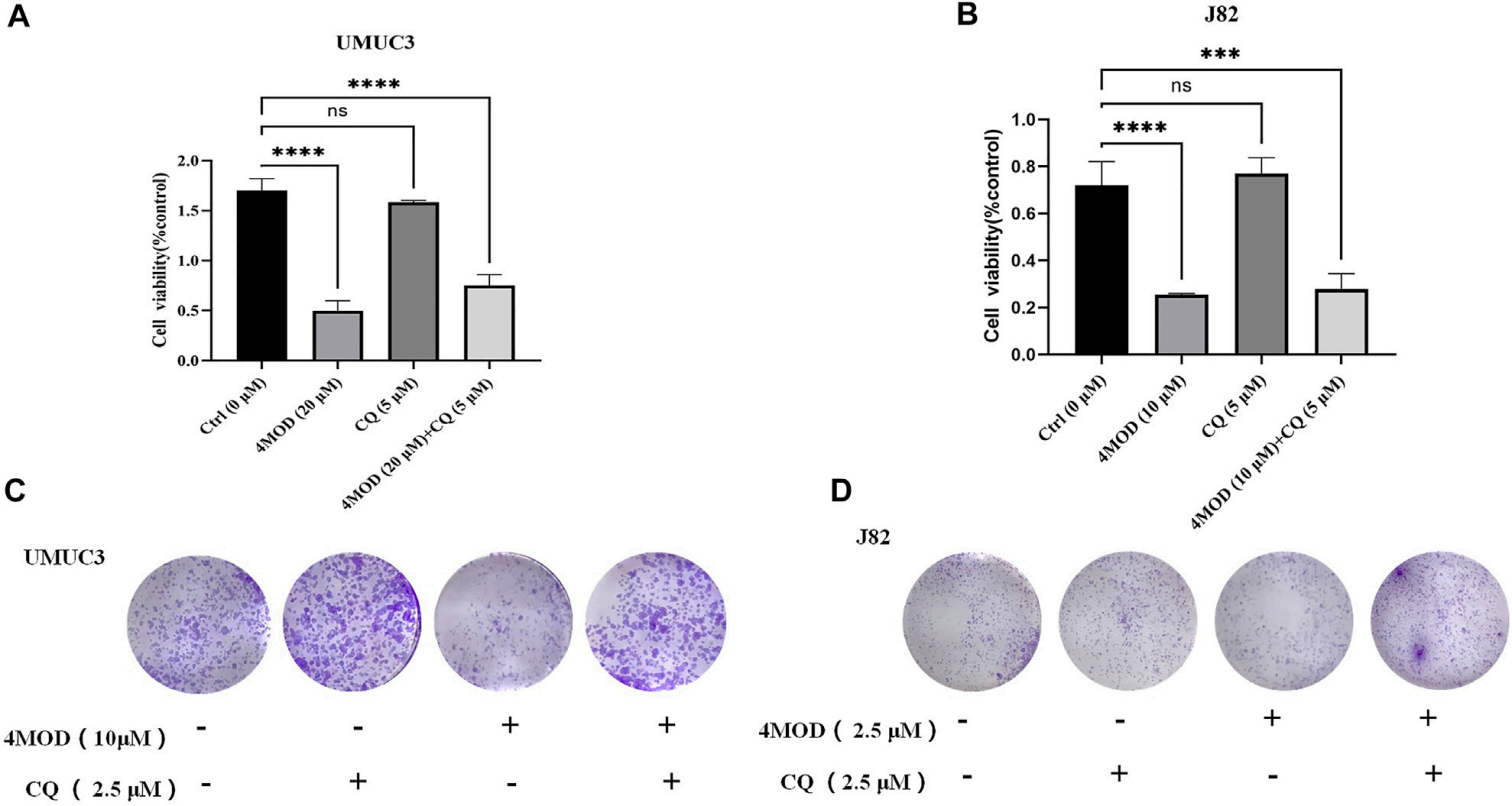
Antagonism action of combination of 4MOD with CQ on proliferation and colony formation in BC Cells **(A–B)** Cell viability was assessed after treatment of 4MOD alone or 4MOD combined with CQ for 72 h **(C–D)** UMUC3 and J82 cells were treated for 7 days with 4MOD combined with CQ and then stained with crystal violet to allow colony counting.

Chloroquine (CQ) first approved for medical use in 1949, have also been widely reported as potential anticancer agents for it can destroy the function of the lysosome and inhibit autophagy (Ferreira et al., 2021). It is one of the classic experiments for observing autophagy flux that the increase in LC3-II protein before and after CQ treatment reflects the amount of autophagosomes degraded by lysosomes. CCK8 assay was utilized to examine the antagonism effect of combining 4MOD with the CQ. As shown in Figures 5A,B, 20 µM 4MOD alone significantly inhibited the proliferation of J82 and UMUC3 cells, while the anti-proliferative effect of 4MOD could be rescued when combined with 5 µM CQ. We next examined the anti-cloning effect of the combination of 4MOD and CQ. Consistent with CCK8 assay, we observed that CQ significantly antagonized the anti-cloning effect of 4MOD (Figures 5C,D). Taken together, these data recognize that inhibition of autophagy may be an effective strategy to increase the anticancer activity of 4MOD in bladder cancer.

### Akt/ERK Signaling Pathways Participate in the Inhibitory Effect of 4MOD on BC Cells Growth

**FIGURE 6:**
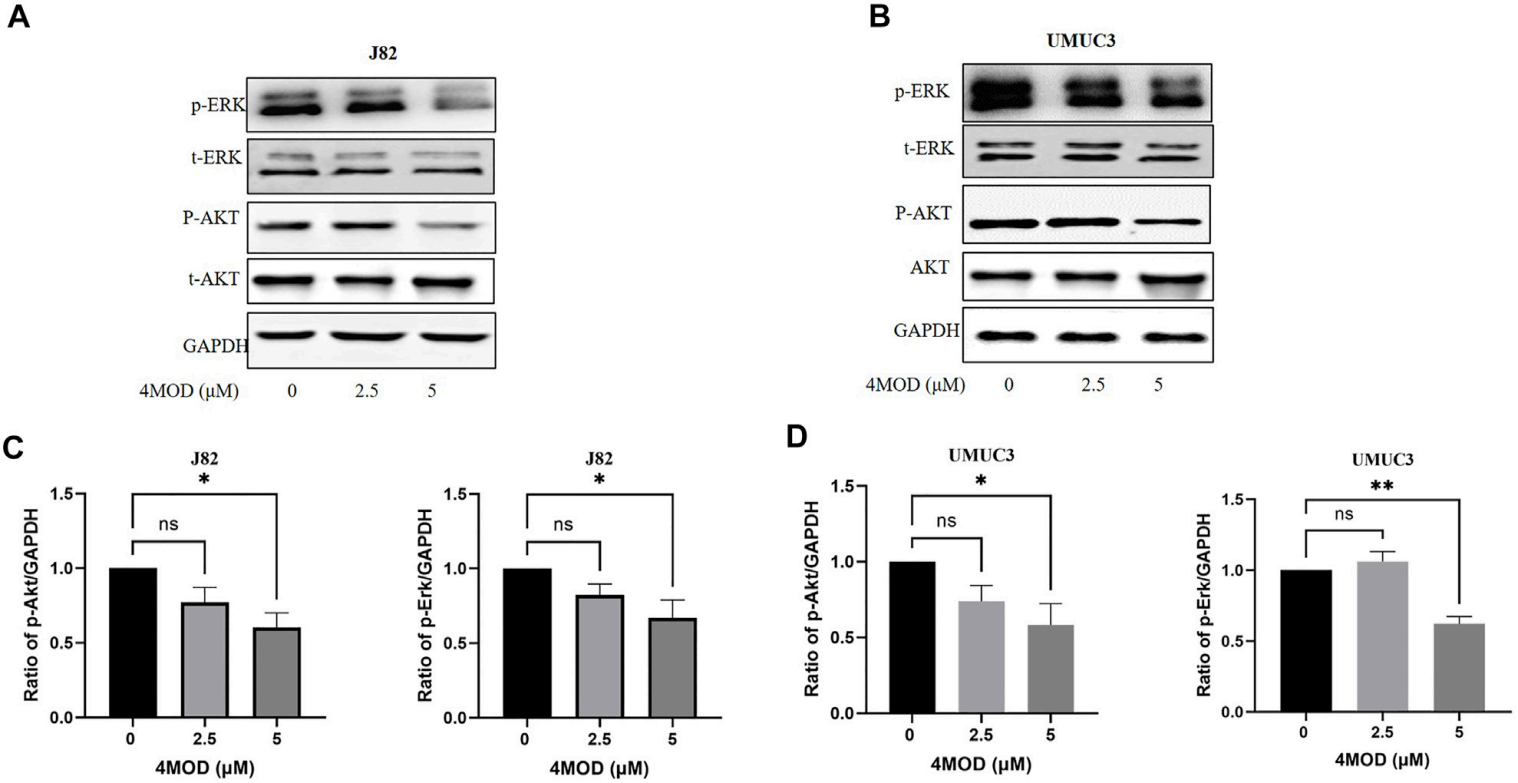
4MOD inhibits Akt/ERK signaling in BC cells **(A–B)** The UMUC3 andJ82 cells were treated with 0–5.0 μM 4MOD for 72 h 4MOD or DMSO. Western blot analysis was used to examine total (t) and phosphorylated (p) forms of Akt and Erk, GAPDH was included as a loading control **(C-D)** Relative levels of phosphorylated Akt, Erk are shown as means ± SD, n = 3, **p < 0.01, *p < 0.05.

Given that Akt/ERK signaling pathways, as critical pathways for tumorigenesis, are key intracellular mediators of cell survival and proliferation signals (Cao et al., 2019). Investigative Akt/ERK signaling during 4MOD treatment are meaningful for us to elaborate its anticancer mechanism. Thus, we explored whether 4MOD induced autophagy by regulating Akt/ERK signaling pathways in the BC cells. As shown in Figures 6A–D, 4MOD diminished the phosphorylation of Akt and/or Erk1/2 while the protein levels of total Erk1/2 and Akt had no significant change in the J82 and UMUC3 cells.

### 4MOD Attenuates BC Progression Through Inhibiting AKT/ERK Signaling *in vitro*

**FIGURE 7:**
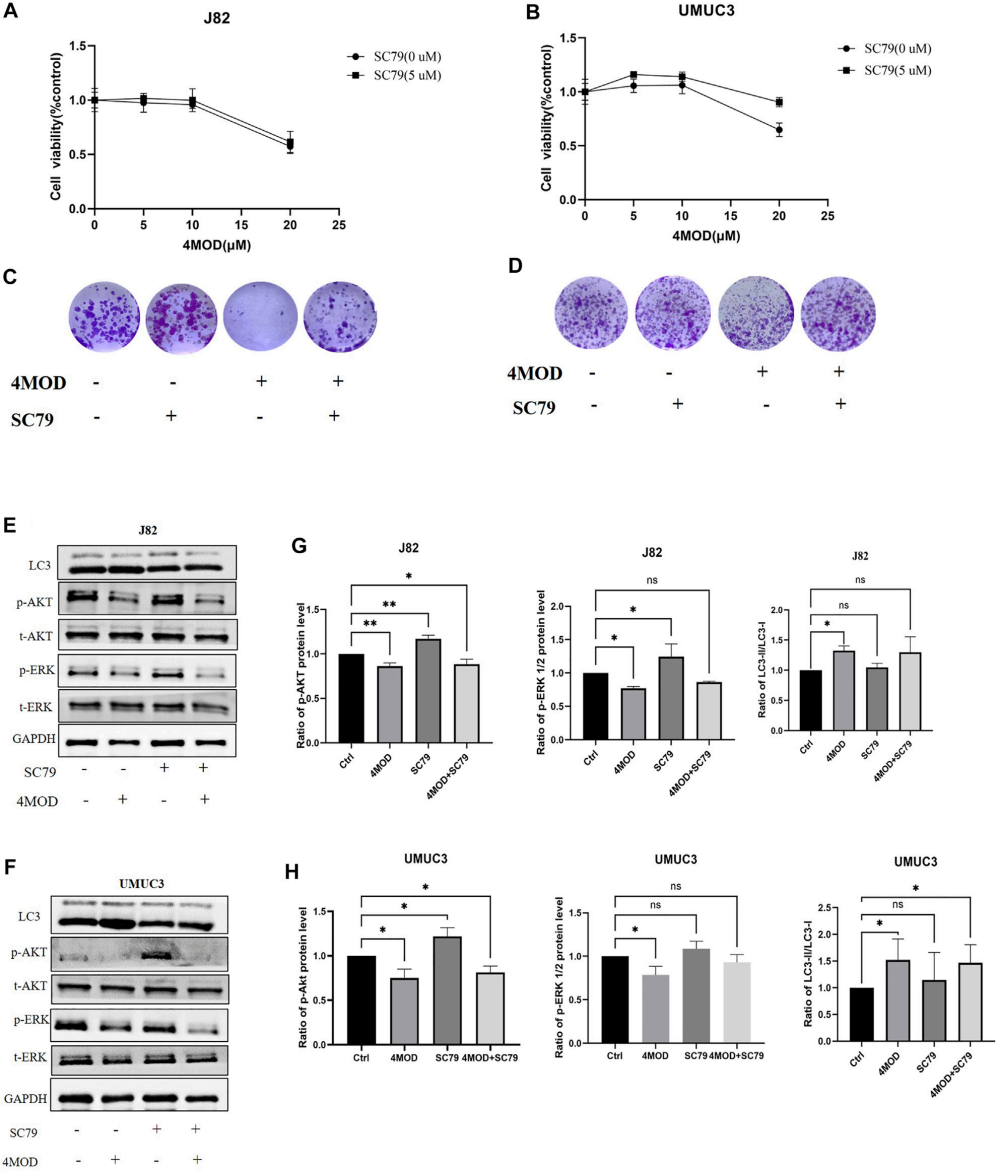
4MOD attenuates BC progression through inhibiting AKT/ERK signaling *in vitro* **(A–B)** Cell viability was assessed after treatment of 4MOD alone or 4MOD combined with Akt antagonist SC79 for 72 h **(C–D)** UMUC3 and J82 cells were treated for 7 days with 4MOD combined with SC79 and then stained with crystal violet to allow colony counting **(E–F)** UMUC3 and J82 cells were after treatment of 4MOD alone or 4MOD combined with SC79 for 72 h, and the protein levels of pErk, t-Erk, pAkt, t-Akt, and LC3 were detected by Western blot with GAPDH as a control. **(G, H)** The results of Western blot analysis were quantified by ImageJ software. Data are presented as mean ± SD. Statistic significant differences were indicated: *p < 0.05, **p < 0.01.

To further determine whether inhibiting Akt/ERK signaling is essential for 4MOD to exert its anti-tumor effect on BC cells, we added Akt specific agonist SC79 in combination with 4MOD in J82 and UMUC3 cells, As shown in Figures 7A,B, 4MOD and SC79 have shown varying degrees of antagonistic effect, the ability of 4MOD to inhibit cancer cell proliferation was reduced after adding SC79. We next observed that when SC79 and 4MOD treated J82 and UMUC3 cells simultaneously, the anti-cloning effect of 4MOD could be reversed (Figures 7C,D). As shown in Figures 7E,F, 4MOD treatment alone inhibited AKT and ERK strongly in both J82 and UMUC3. while SC79 could rescue the 4MOD-inhibited phosphorylation of AKT and ERK when cells were treated with SC79 combination with 4MOD. Meanwhile, the expression ratio of light chain 3-II (LC3-II)/LC3-I were increased in the J82 and UMUC3 cells when the treatment of 4MOD, and SC79 could reverse this phenotype in the UMUC3 while without affecting J82 cells. Figures 7G,H showed the statistical analysis of protein quantifications (*p < 0.05). Together these suggest that 4MOD attenuates BC progression through inhibiting AKT/ERK signaling *in vitro*.

## Discussion

Bladder cancer is one of the malignancies in the urinary system. The incidence of bladder cancer ranks ninth among global malignant tumors, and second among urinary system tumors (Siegel et al., 2018). 4MOD is a natural flavonoid. Our previous study found that 4MOD can block cell cycle and promote apoptosis in astroglioma cells, thus inhibiting tumor growth (Park et al., 2016). The latest research showed that 4MOD has anti-osteosarcoma effect (Park et al., 2016). Nonetheless, the role and the underlying molecular mechanisms of 4MOD in the development of BC cells is not reported. In this study, we identified the antitumor potential of 4MOD on BC cells.

It was known that tumor cell proliferation and apoptosis are important processes in tumorigenesis and development. Inhibiting the proliferation of tumor cells and inducing their apoptosis is the focus of research on the anti-tumor activity of natural products (Jin and Shi, 2016). Our results show that 4MOD can inhibit BC cells proliferation and induce cell apoptosis in a dose-dependent manner by CCK8 assay, colony formation, TUNEL and flow cytometry detection of apoptosis. Furthermore, 4MOD also represses invasion and migration of BC cells. These results show that 4MOD has research value in BC and can be used as a candidate drug for its clinical treatment.

Autophagy, is a key lysosomal degradation pathway, which involves in BC development and progression (Chen et al., 2018). Ojha (Ojha et al., 2014) et al. found that LC3 was significantly increased in both high-grade and low-grade urothelial cancer specimens, and the increase was more significant in high-grade urothelial cancer specimens. In our assays, we found that the LC3 gene expression level significantly increased after treatment with 4MOD by using the 2^−ΔΔCT^ method and normalization to 18s RNA as a reference. Furthermore, Western blot results showed that the expression of LC3 and Beclin 1 was increased after treatment with 4MOD. We further found that an antagonistic effect exists on proliferation and colony formation when combination of 4MOD with CQ in BC Cells. These data collectively demonstrate that 4MOD can induce autophagy to exert anticancer effect on BC.

In this study, we demonstrated that 4MOD downregulated the phosphorylation of both Akt and Erk1/2. Activation of Akt and ERK is crucial for tumor growth and resistance on anticancer drugs (McCubrey et al., 2007; Wang et al., 2009; Liu et al., 2014). We further found varying degrees of antagonistic effects on the proliferation and colony formation of J82 and UMUC3 cells treated with 4MOD and SC79. These results showed that Akt/ERK signaling pathway is necessary for 4MOD to exert its anti-tumor effect.

## Conclusion

In summary, the present study demonstrates the anti-proliferative potential of 4MOD on BC cells by inducing autophagy and inhibiting Akt/ERK signaling pathways. This study is the first to evaluate the antitumor action of 4MOD on BC cells. Our findings suggest that 4MOD could be a new treatment for BC.

## Data Availability

The datasets presented in this study can be found in online repositories. The names of the repository/repositories and accession number(s) can be found in the article/Supplementary Material.
