# Supplementary material for: 4-Methoxydalbergione Inhibits Bladder Cancer Cell Growth via Inducing Autophagy and Inhibiting Akt/ERK Signaling Pathway
Source: Front Mol Biosci. 2022 Feb 16;8:789658. doi: 10.3389/fmolb.2021.789658 (PMC8888913; doi:10.3389/fmolb.2021.789658)
Supplement: Supplementary file 1 [file Table1.DOCX]

**Western blot description**

**The expression of β-actin: Data is shown as 3 independent experiments.**

**
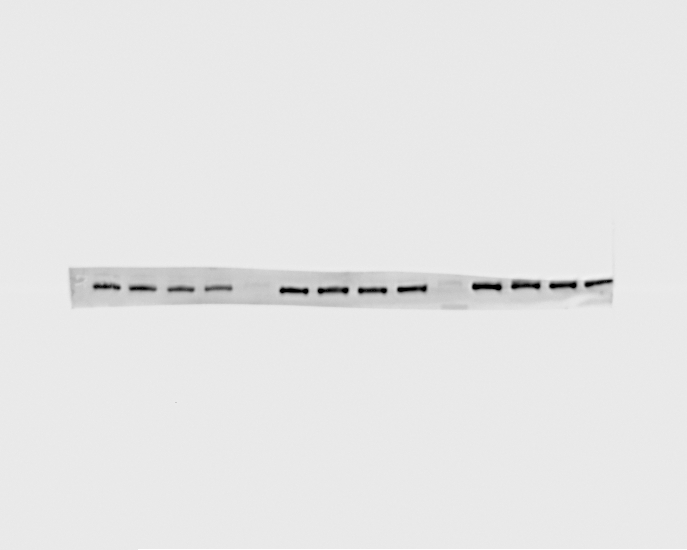
1.**

The J82 (Four in the front) and UMUC3 (four in the middle) cells were treated with 0, 2.5, 5, 10 and 0, 5, 10, 20 μM 4MOD for 72 h. (The last four are other results)


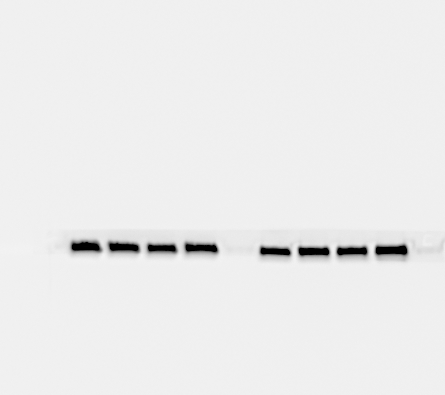
**2.**

The J82 (Four in the front) and UMUC3 (four in the back) cells were treated with 0, 2.5, 5, 10 and 0, 5, 10, 20 μM 4MOD for 72 h.


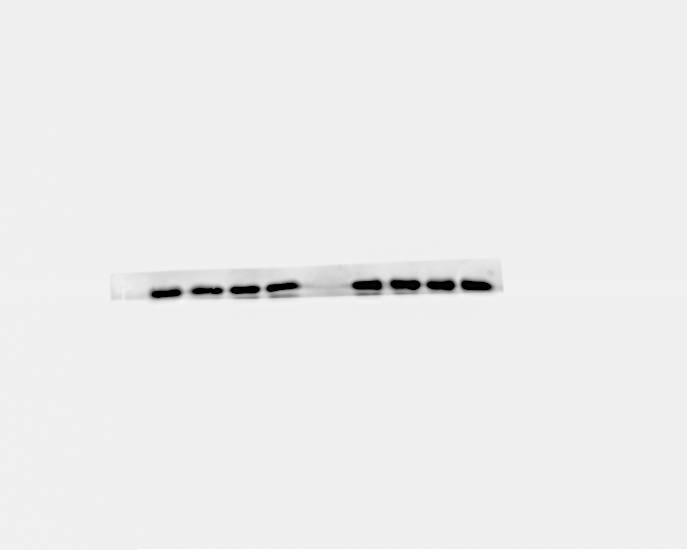
**3.**

The J82 (Four in the front) and UMUC3 (four in the back) cells were treated with 0, 2.5, 5, 10 and 0, 5, 10, 20 μM 4MOD for 72 h.

**The expression of LC3: Data is shown as 3 independent experiments.**


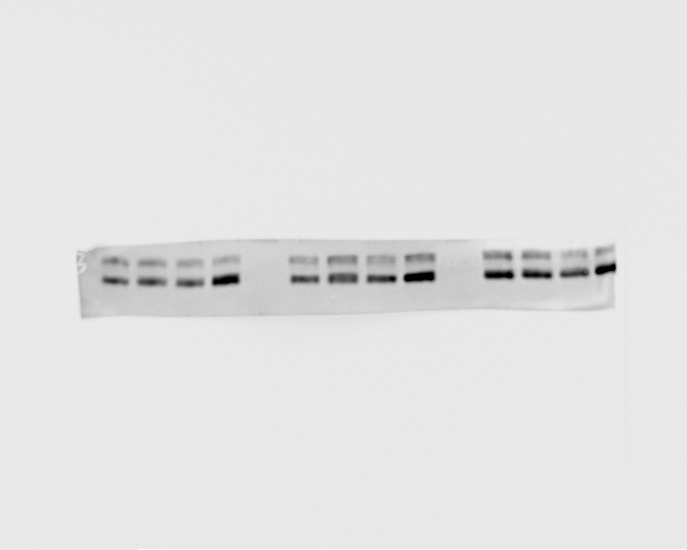
**1.**

The J82 (Four in the front) and UMUC3 (four in the middle) cells were treated with 0, 2.5, 5, 10 and 0, 5, 10, 20 μM 4MOD for 72 h. (The last four are other results)

**2.**


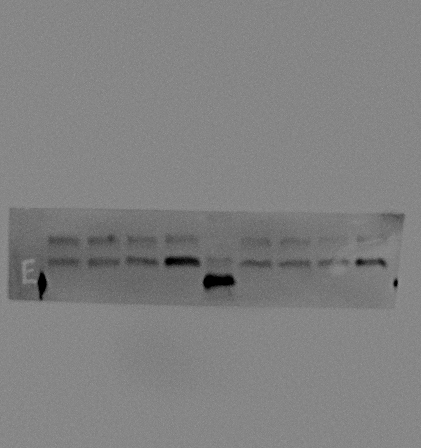


The J82 (Four in the front) and UMUC3 (four in the back) cells were treated with 0, 2.5, 5, 10 and 0, 5, 10, 20 μM 4MOD for 72 h.

**
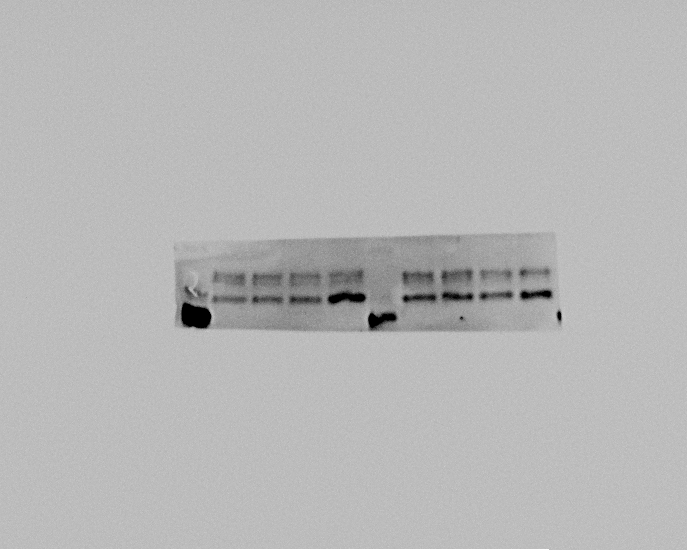
**3.

The J82 (Four in the front) and UMUC3 (four in the back) cells were treated with 0, 2.5, 5, 10 and 0, 5, 10, 20 μM 4MOD for 72 h.


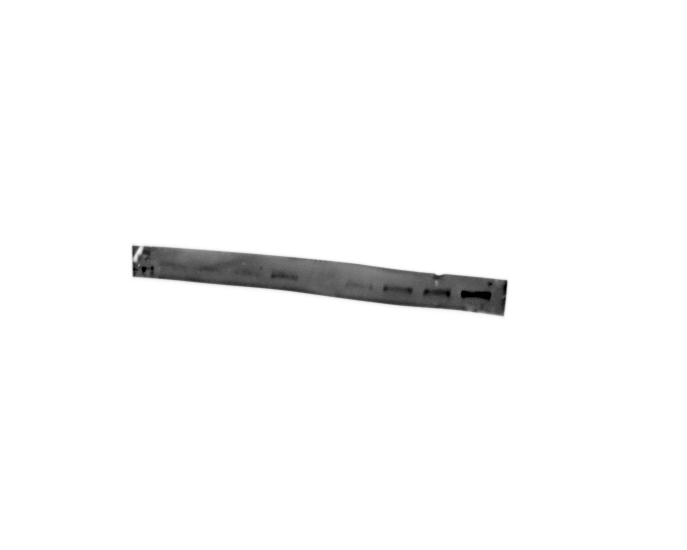
**The expression of beclin 1: Data is shown as 3 independent experiments.**

The J82 (Four in the front) and UMUC3 (four in the back) cells were treated with 0, 2.5, 5, 10 and 0, 5, 10, 20 μM 4MOD for 72 h.


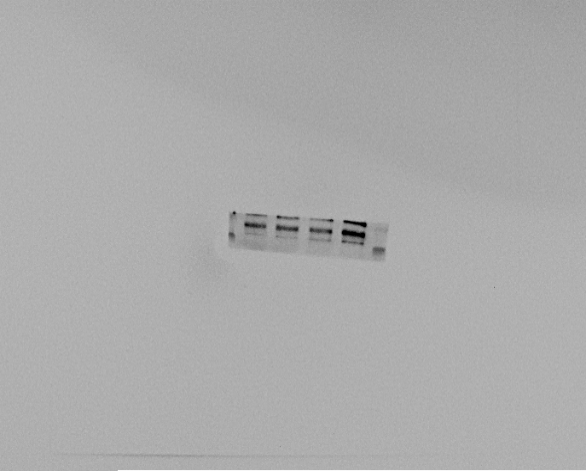


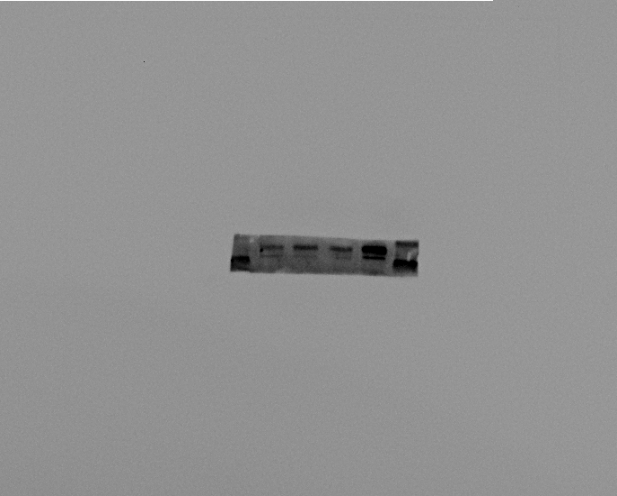


The J82 (first) and UMUC3 (back) cells were treated with 0, 2.5, 5, 10 and 0, 5, 10, 20 μM 4MOD for 72 h.


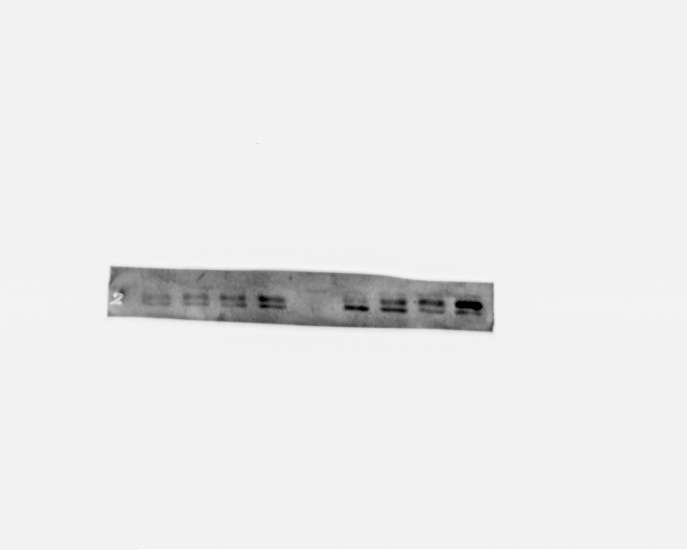


The J82 (Four in the front) and UMUC3 (four in the back) cells were treated with 0, 2.5, 5, 10 and 0, 5, 10, 20 μM 4MOD for 72 h.

**The expression of GAPDH: Data is shown as 3 independent experiments.**


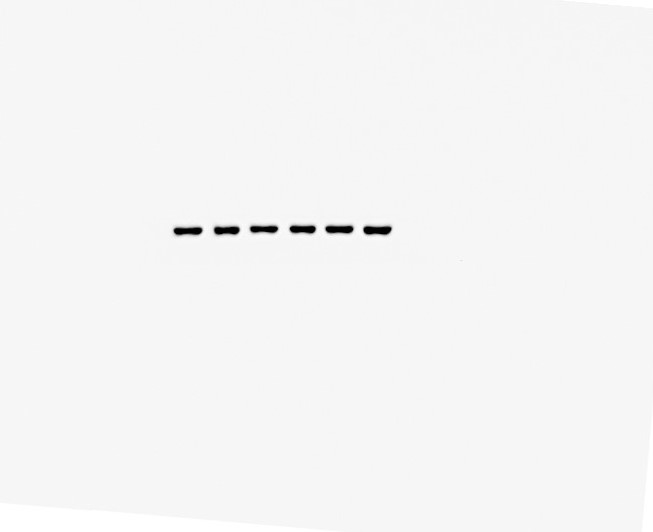


The J82 (three in the front) and UMUC3 (three in the back) cells were treated with 0, 2.5, 5 μM 4MOD for 72 h.


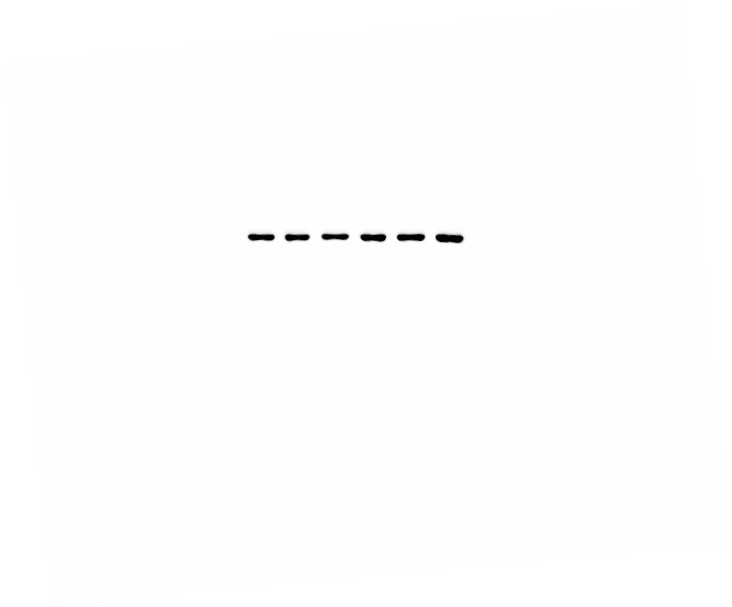


The J82 (three in the front) and UMUC3 (three in the back) cells were treated with 0, 2.5, 5 μM 4MOD for 72 h.


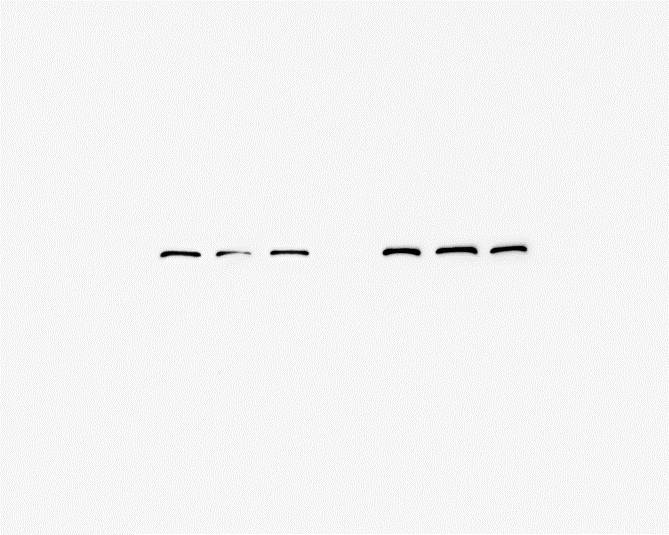


The J82 (three in the front) and UMUC3 (three in the middle) cells were treated with 0, 2.5, 5 and 0, 2.5, 5 μM 4MOD for 72 h.

**The expression of p-Akt: Data is shown as 3 independent experiments.**


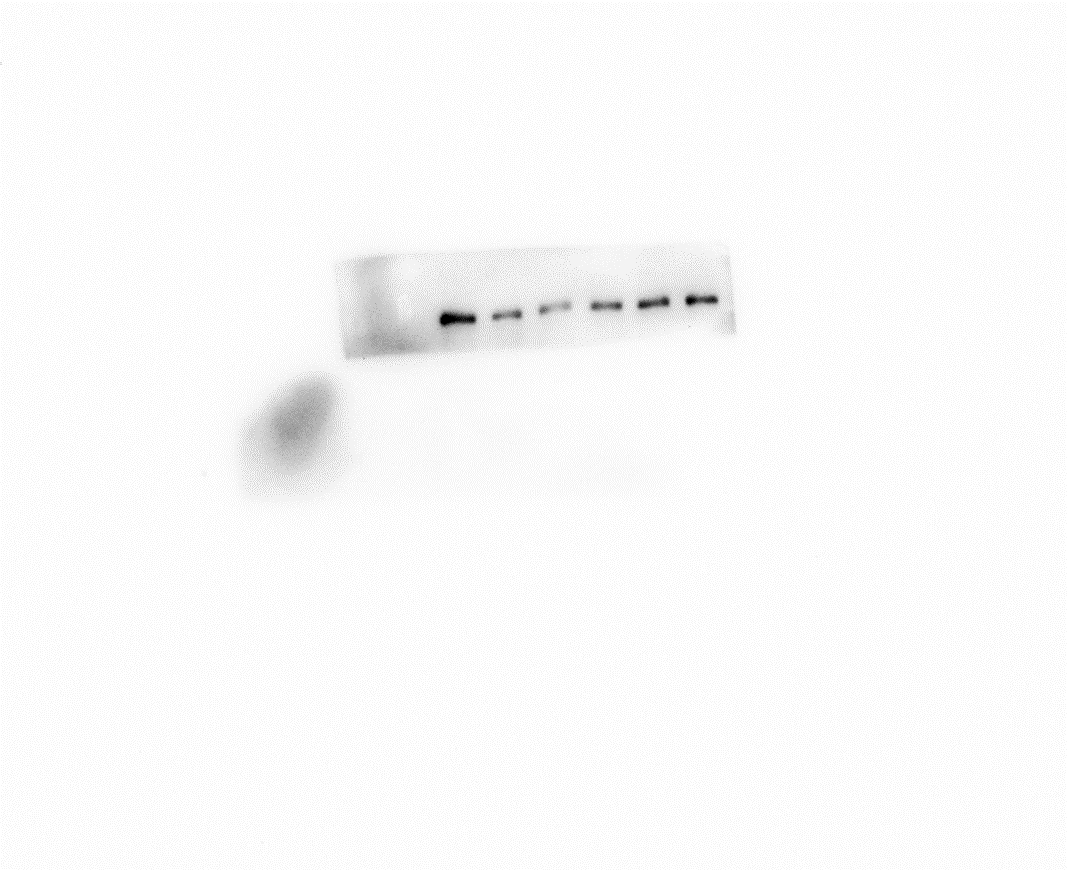


The J82 (three in the front) and UMUC3 (three in the back) cells were treated with 0, 2.5, 5 μM 4MOD for 72 h.


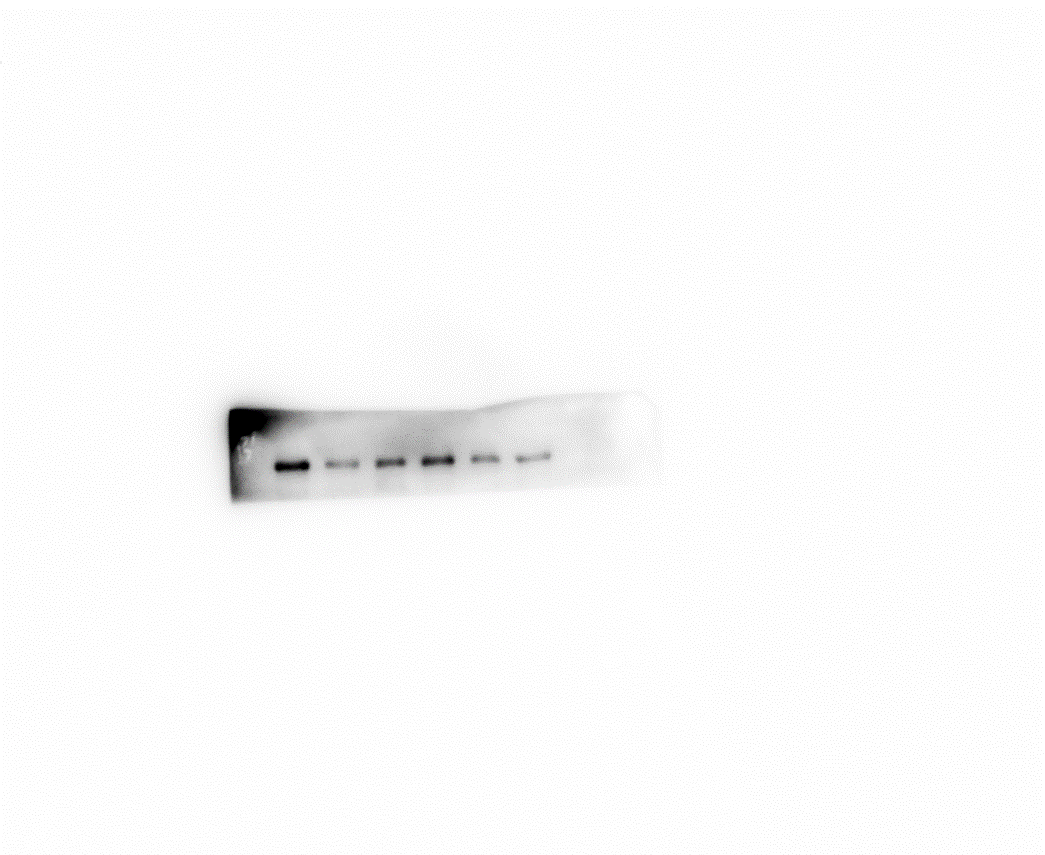


The J82 (three in the front) and UMUC3 (three in the back) cells were treated with 0, 2.5, 5 μM 4MOD for 72 h.





The J82 (three in the front) and UMUC3 (three in the back) cells were treated with 0, 2.5, 5 μM 4MOD for 72 h.

**The expression of p-ERK: Data is shown as 3 independent**

**

**

The J82 (three in the front) and UMUC3 (three in the back) cells were treated with 0, 2.5, 5 μM 4MOD for 72 h.

**

**

The J82 (three in the front) and UMUC3 (three in the back) cells were treated with 0, 2.5, 5 μM 4MOD for 72 h.





The J82 (three in the front) and UMUC3 (three in the back) cells were treated with 0, 2.5, 5 μM 4MOD for 72 h.

**
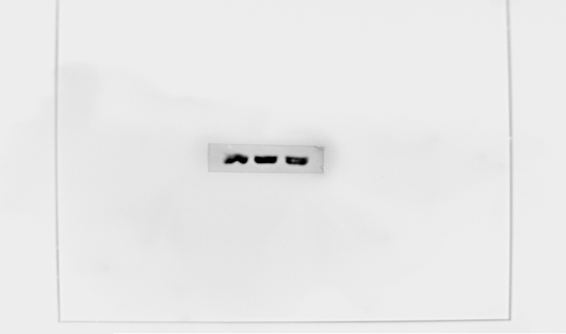

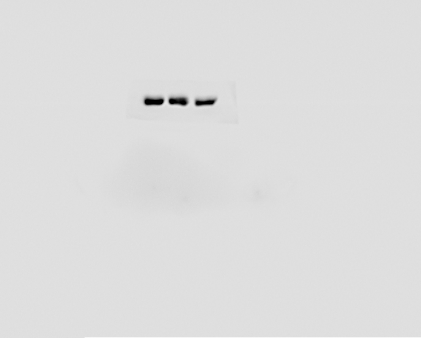
**
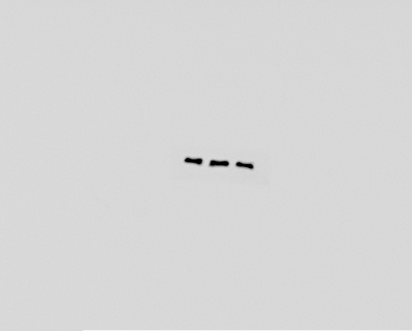
**The expression of t-Akt: Data is shown as 3 independent**

The J82 cell was treated with 0, 2.5, 5 μM 4MOD for 72 h.


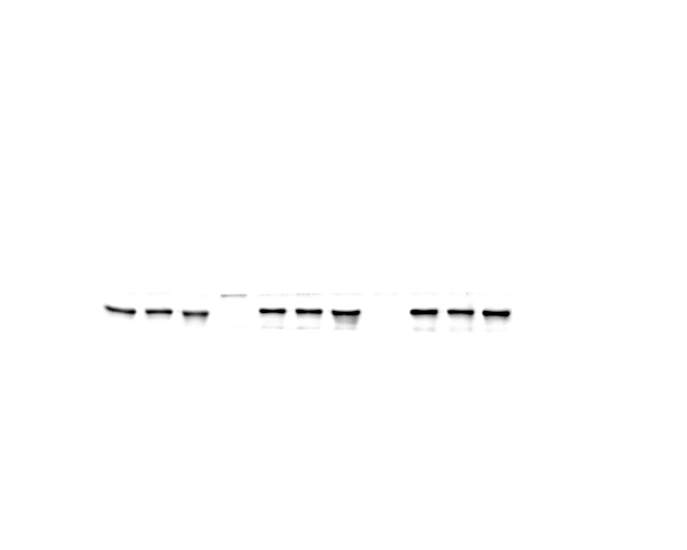


The UMUC3 cell was treated with 0, 2.5, 5 μM 4MOD for 72 h.

**The expression of t-ERK: Data is shown as 3 independent**

**
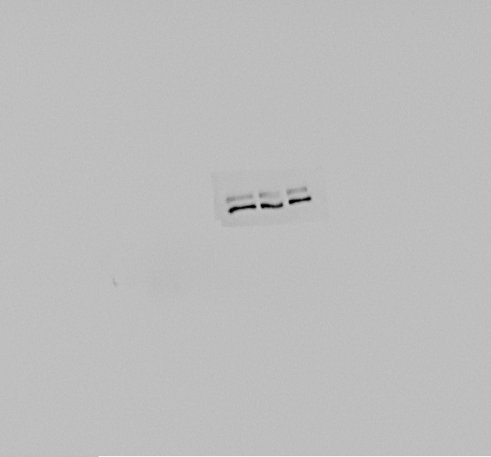
**
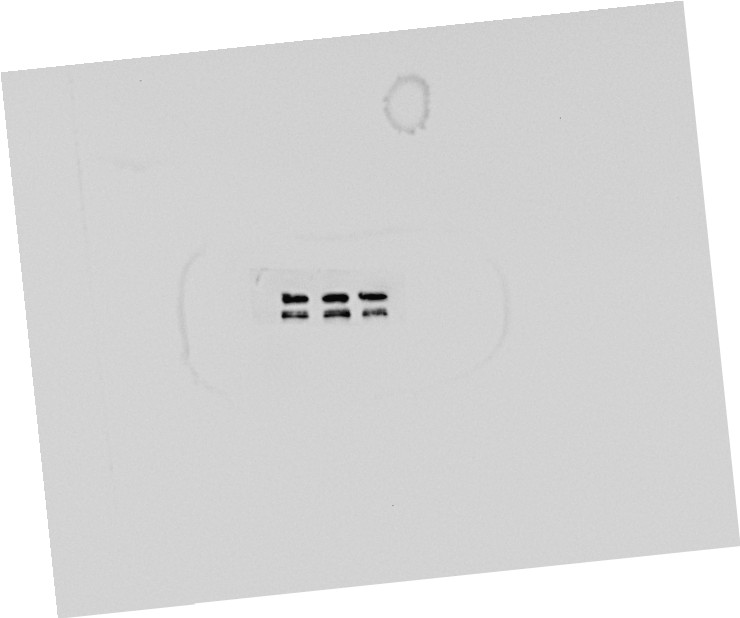

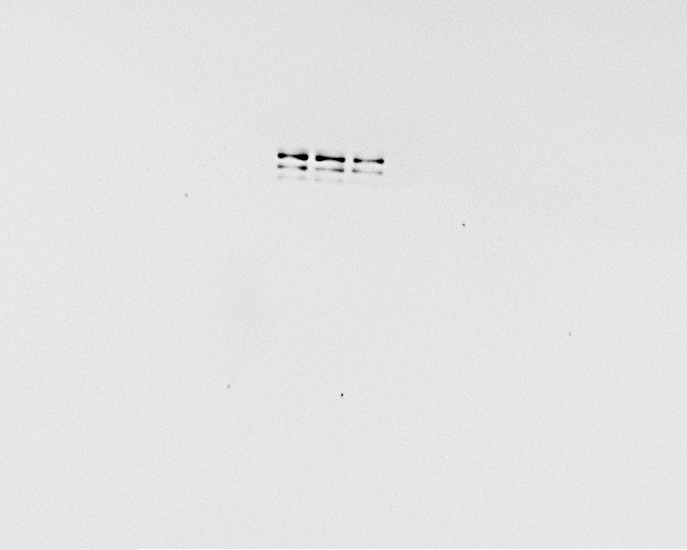


The J82 cell was treated with 0, 2.5, 5 μM 4MOD for 72 h.


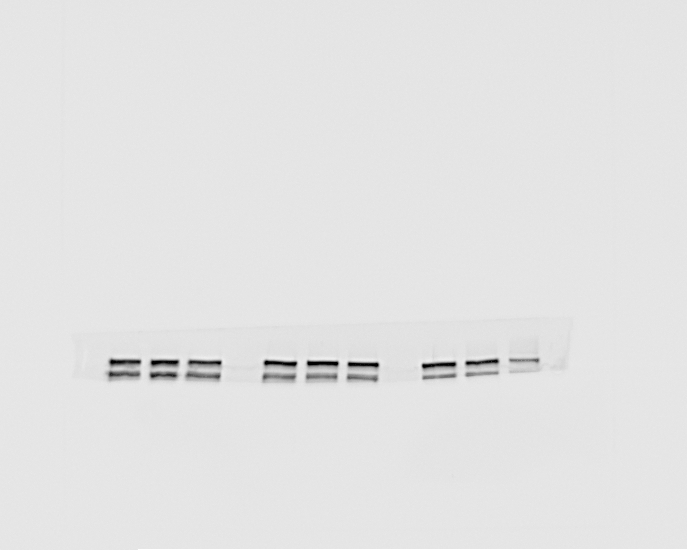


The UMUC3 cell was treated with 0, 2.5, 5 μM 4MOD for 72 h.
