# Supplementary material for: 4-Methoxydalbergione Inhibits Bladder Cancer Cell Growth via Inducing Autophagy and Inhibiting Akt/ERK Signaling Pathway
Source: Front Mol Biosci. 2022 Feb 16;8:789658. doi: 10.3389/fmolb.2021.789658 (PMC8888913; doi:10.3389/fmolb.2021.789658)
Supplement: Supplementary file 2 [file Table8.DOCX]

Flow cytometry—fcs

<https://www.jianguoyun.com/p/DS_mulYQ64D1CRjCz5IE>

Scratch assays

<https://www.jianguoyun.com/p/DWrz1HgQ64D1CRjHz5IE>

Transwell assays

<https://www.jianguoyun.com/p/DQBZUP0Q64D1CRjKz5IE>

TUNEL

<https://www.jianguoyun.com/p/DZp6AVkQ64D1CRjNz5IE>

Statistical result

<https://www.jianguoyun.com/p/DXsECK0Q64D1CRjP0pIE>
