# Supplementary material for: 4-Methoxydalbergione Inhibits Bladder Cancer Cell Growth via Inducing Autophagy and Inhibiting Akt/ERK Signaling Pathway
Source: Front Mol Biosci. 2022 Feb 16;8:789658. doi: 10.3389/fmolb.2021.789658 (PMC8888913; doi:10.3389/fmolb.2021.789658)
Supplement: Supplementary file 5 [file Table2.DOCX]

**Transwell**

**J82(0μM, 5μM, 10 μM): Data is shown as 3 independent experiments.**


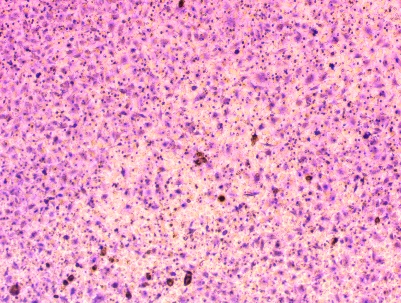


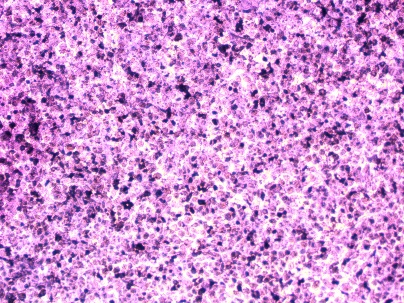
**
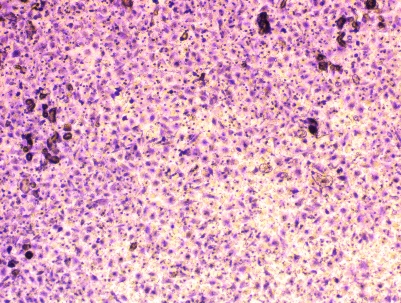
**

The J82 cell was treated with 0 μM 4MOD for 48h

**
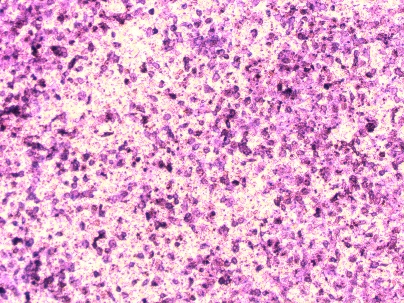

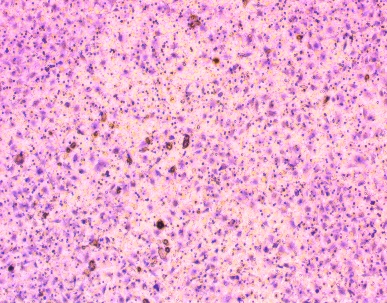
**

**
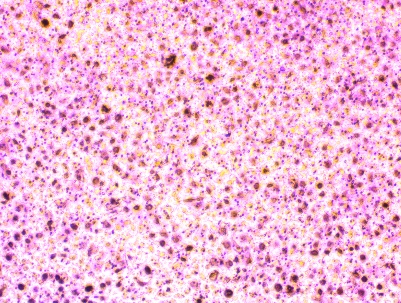
**The J82 cell was treated with 5 μM 4MOD for 48h


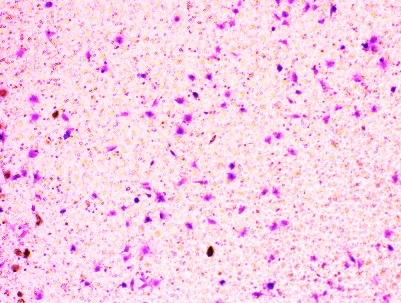

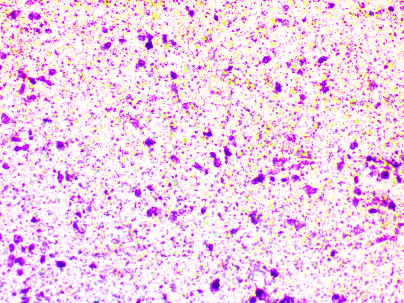

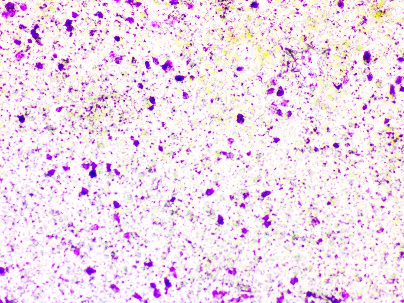


The J82 cell was treated with 10 μM 4MOD for 48h

**UMUC3 (0μM, 5μM, 10 μM): Data is shown as 3 independent experiments.**


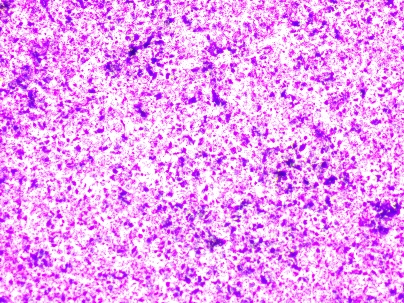

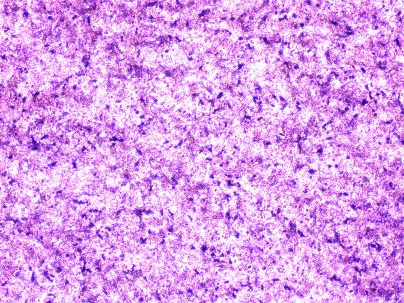

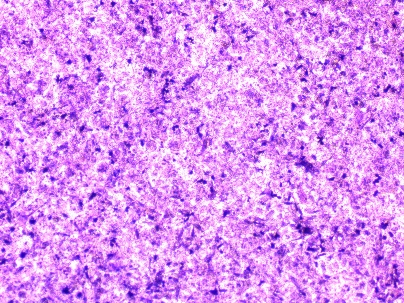


The UMUC3 cell was treated with 0 μM 4MOD for 48h


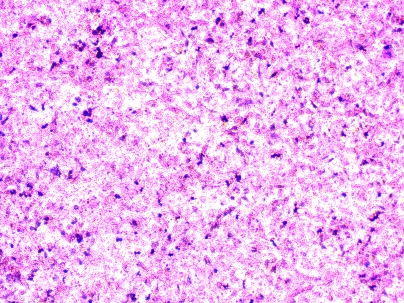

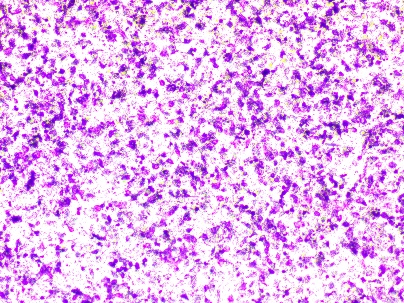

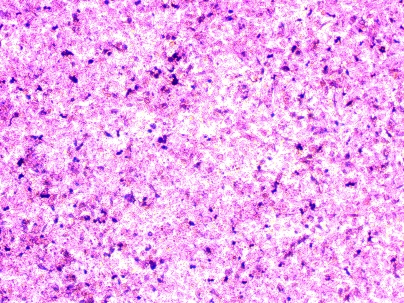


The UMUC3 cell was treated with 5 μM 4MOD for 48h


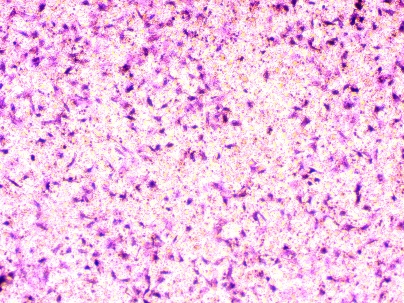

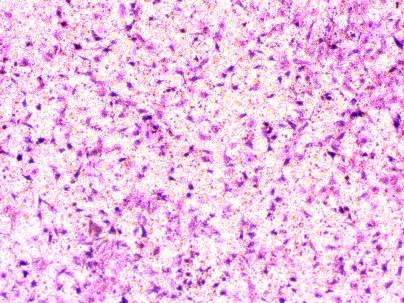

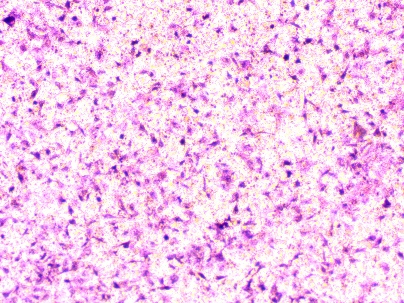


The UMUC3 cell was treated with 10 μM 4MOD for 48h
