# Supplementary material for: 4-Methoxydalbergione Inhibits Bladder Cancer Cell Growth via Inducing Autophagy and Inhibiting Akt/ERK Signaling Pathway
Source: Front Mol Biosci. 2022 Feb 16;8:789658. doi: 10.3389/fmolb.2021.789658 (PMC8888913; doi:10.3389/fmolb.2021.789658)
Supplement: Supplementary file 6 [file Table3.DOCX]

**clonogenic assay**


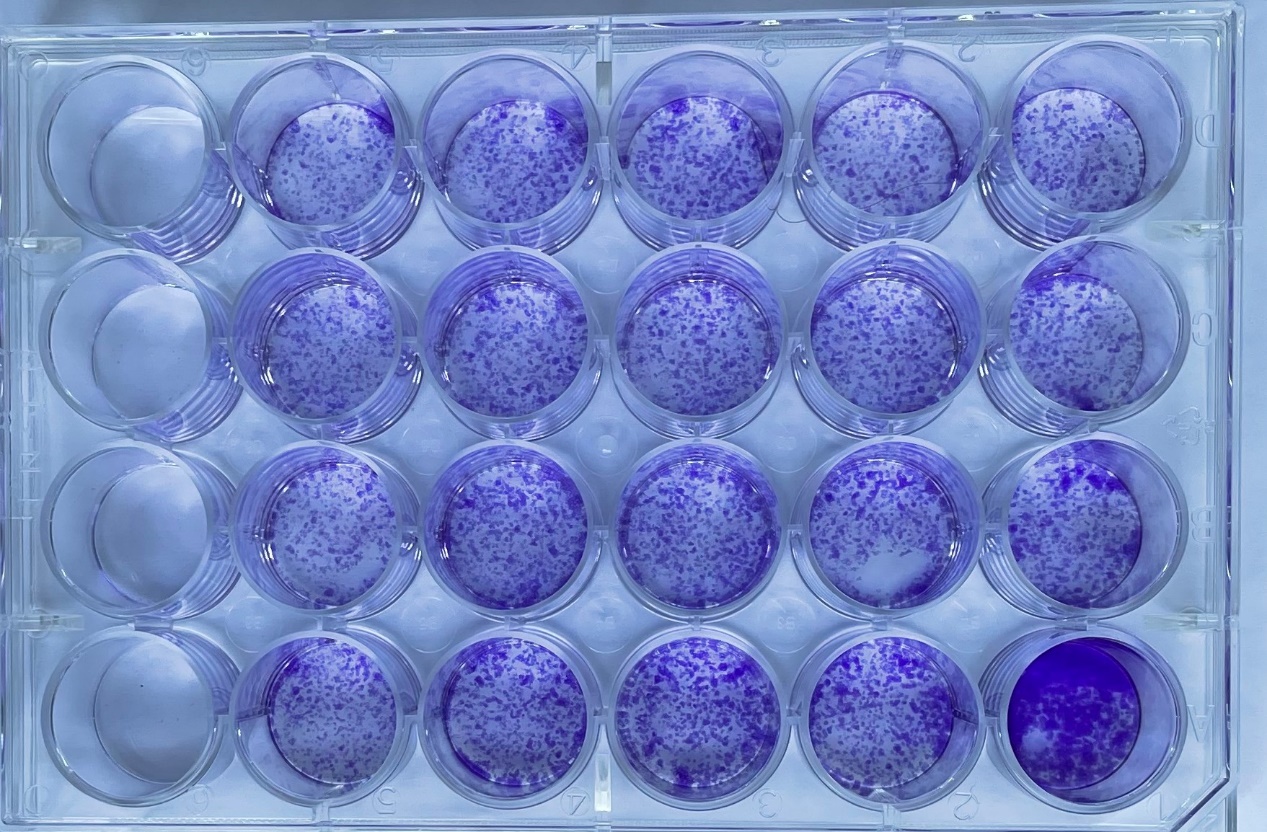


| Blank | 0 μM | 2.5 μM | 5.0 μM | 10.0 μM | 20.0 μM |
| --- | --- | --- | --- | --- | --- |
| Blank | 0 μM | 2.5 μM | 5.0 μM | 10.0 μM | 20.0 μM |
| Blank | 0 μM | 2.5 μM | 5.0 μM | 10.0 μM | 20.0 μM |
| Blank | 0 μM | 2.5 μM | 5.0 μM | 10.0 μM | 20.0 μM |

The UMUC3 cell assessed after 7-day of 4MOD treatment at various concentrations (0-20 μM)


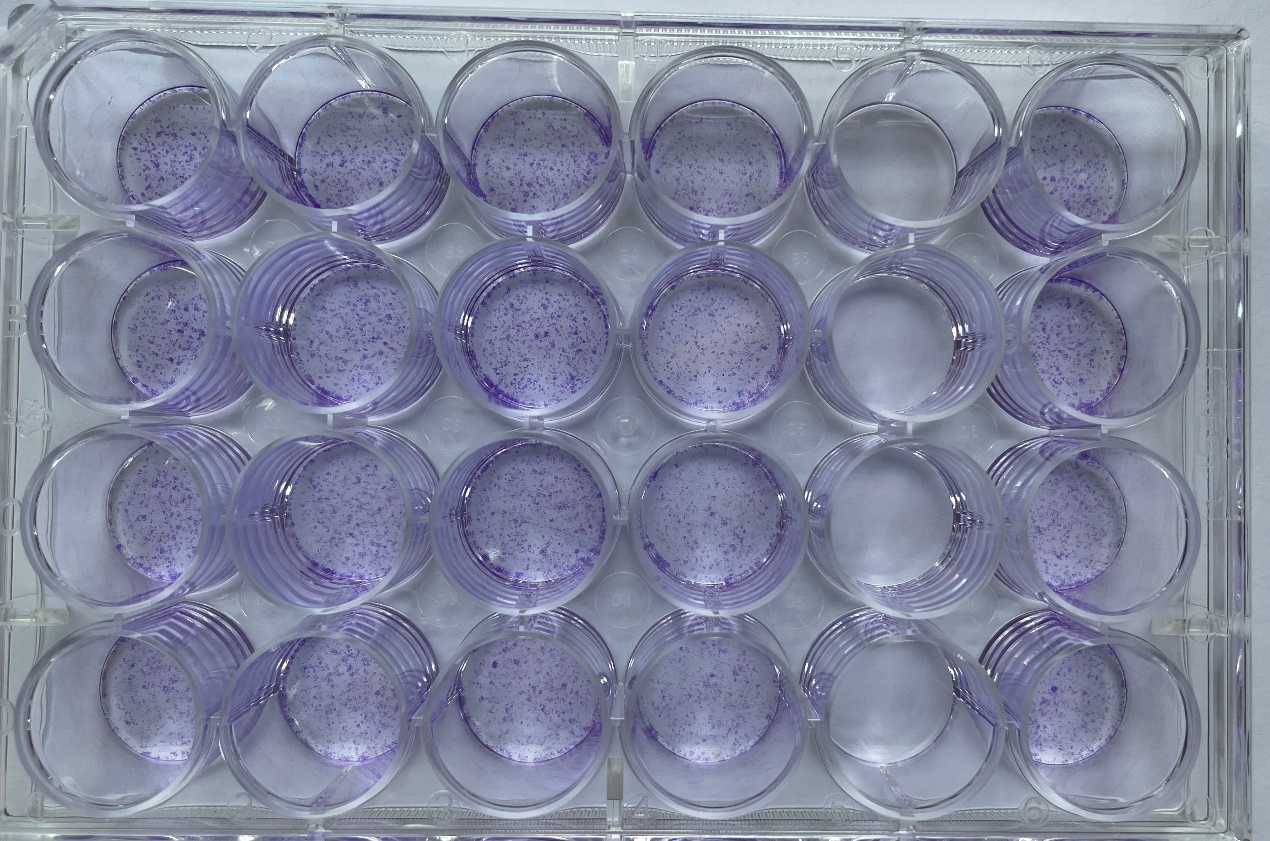


| 0 μM | 2.5 μM | 5.0μM | 10.0 μM | 20.0 μM | blank |
| --- | --- | --- | --- | --- | --- |
| 0 μM | 2.5 μM | 5.0μM | 10.0 μM | 20.0 μM | blank |
| 0 μM | 2.5 μM | 5.0μM | 10.0 μM | 20.0 μM | blank |
| 0 μM | 2.5 μM | 5.0μM | 10.0 μM | 20.0 μM | blank |

The J82 cell assessed after 7-day of 4MOD treatment at various concentrations (0-20 μM)
