# Supplementary figures and images for: 4-Methoxydalbergione Inhibits Bladder Cancer Cell Growth via Inducing Autophagy and Inhibiting Akt/ERK Signaling Pathway
Source: Front Mol Biosci. 2022 Feb 16;8:789658. doi: 10.3389/fmolb.2021.789658 (PMC8888913; doi:10.3389/fmolb.2021.789658)

**Cell apoptosis - flow cytometry**

**UMUC3:**

**U-0 (4MOD 0μM), U-10 (4MOD 10μM); U-20 (4MOD 20μM)**


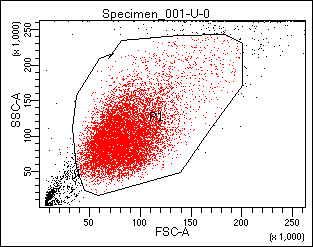

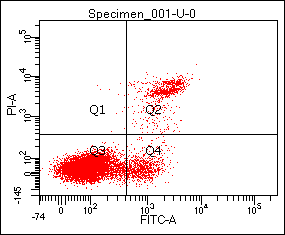

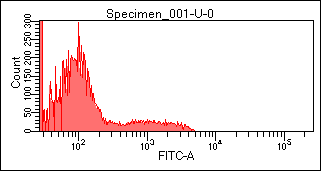

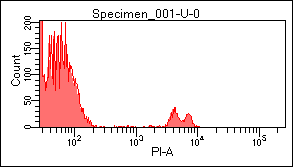


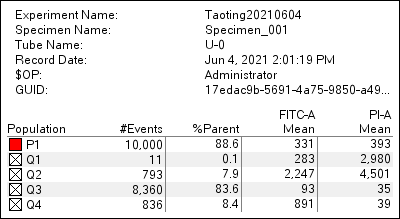


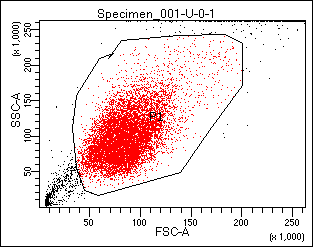

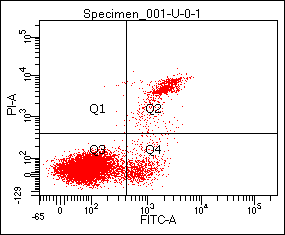

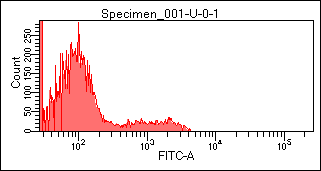

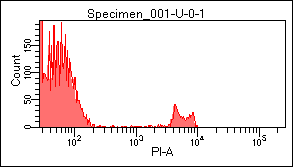

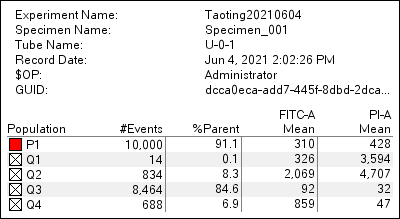

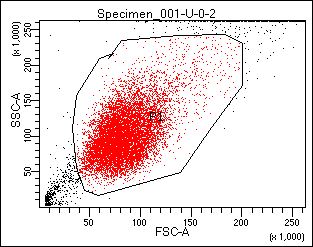

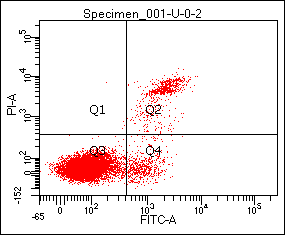

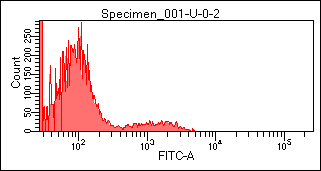

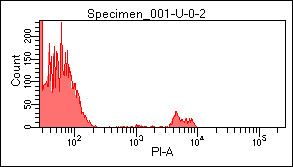

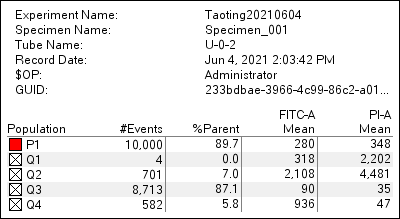

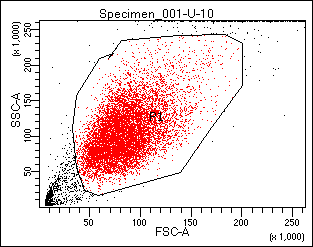

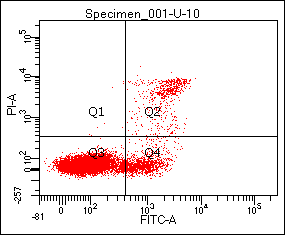

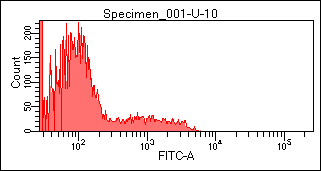

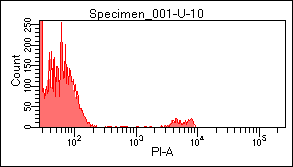

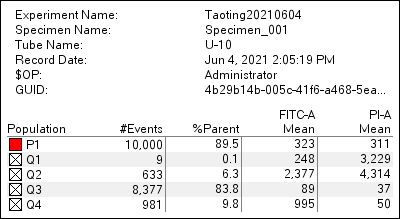

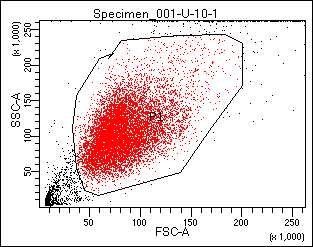

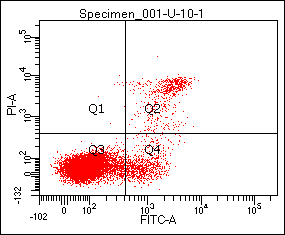

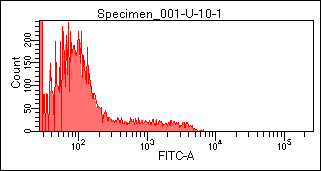

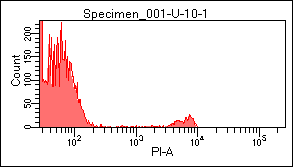

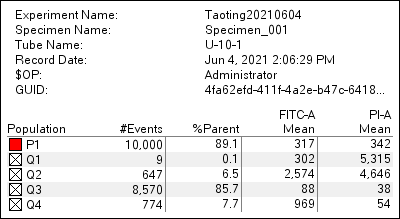

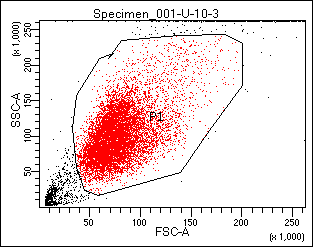

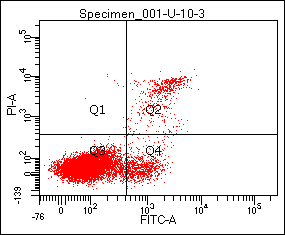

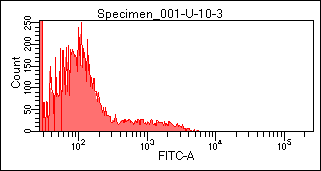

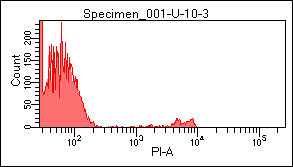

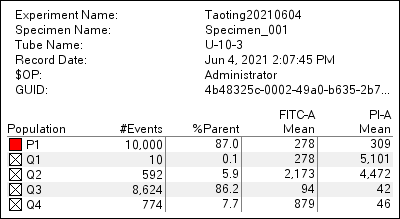

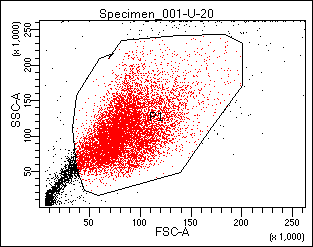

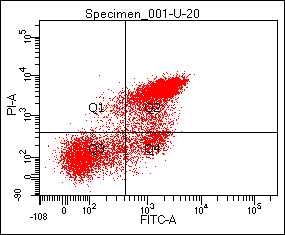

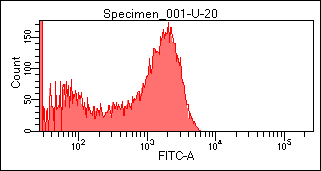

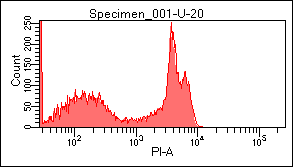

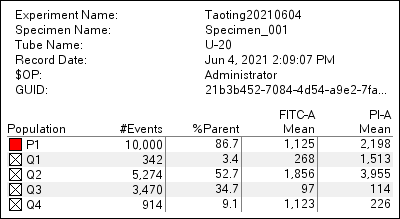

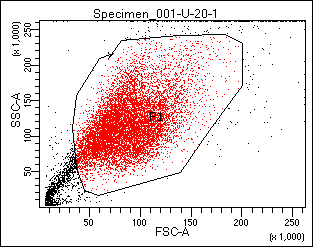

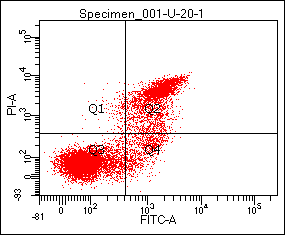

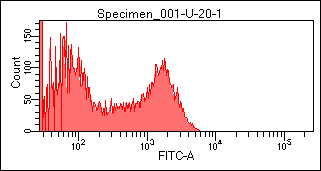

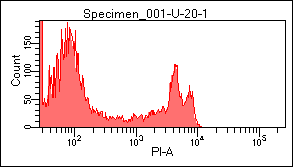

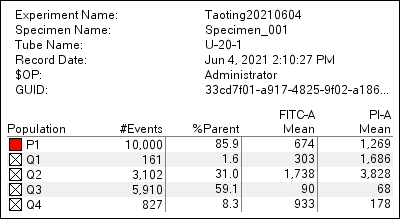

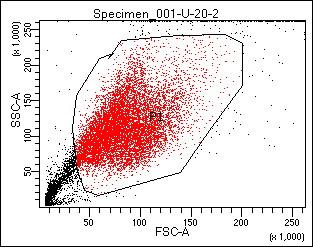

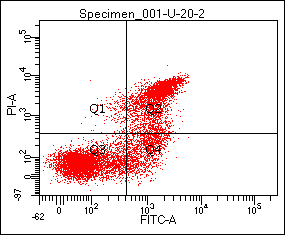

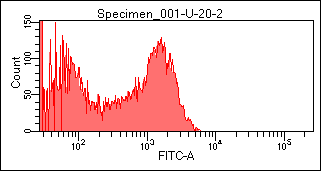

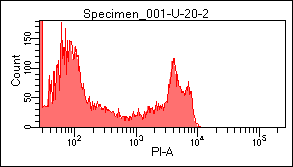

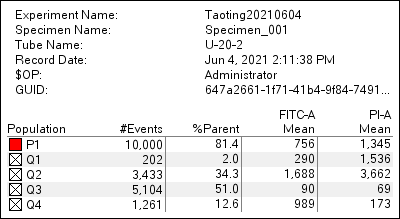


**J82:**

**J-0 (4MOD 0μM), J-10 (4MOD 10μM); J-20 (4MOD 20μM)**


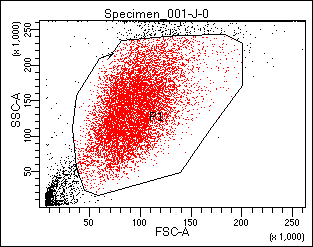

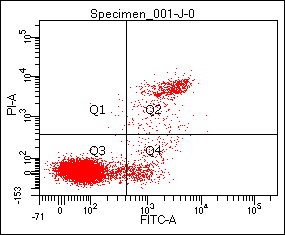

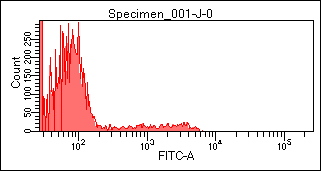

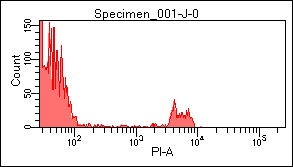

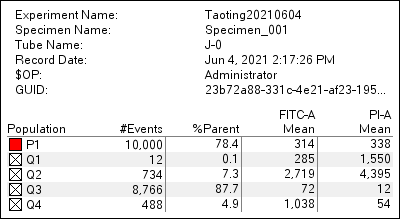

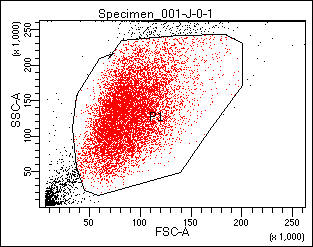

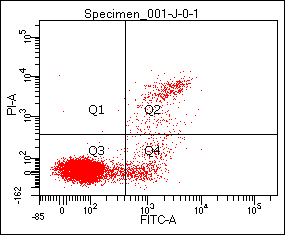

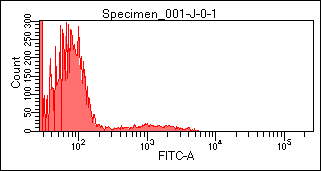

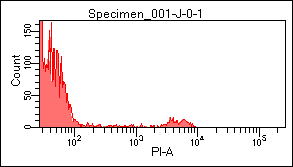

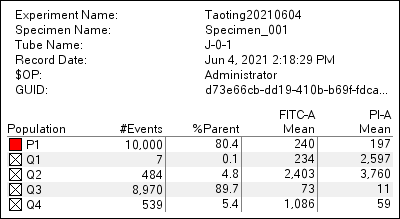

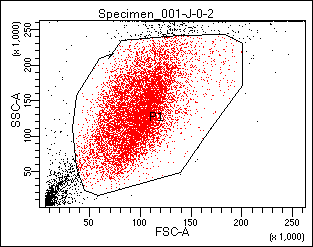

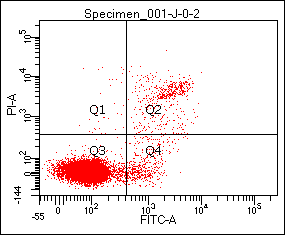

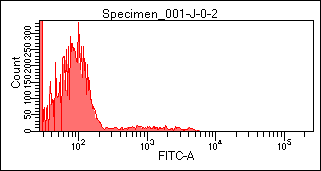

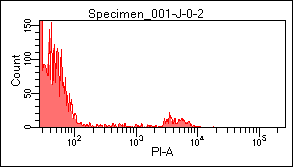

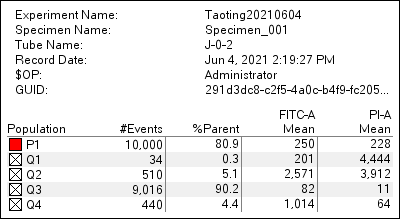

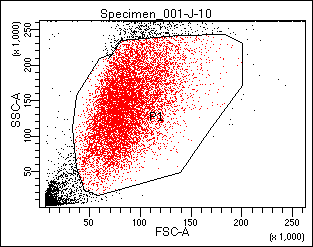

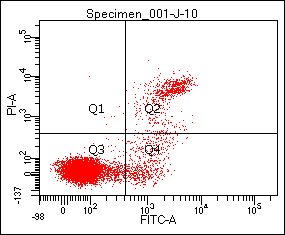

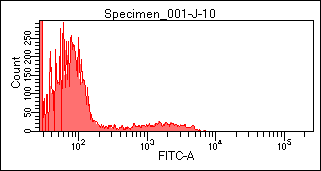

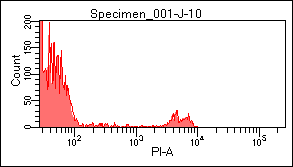

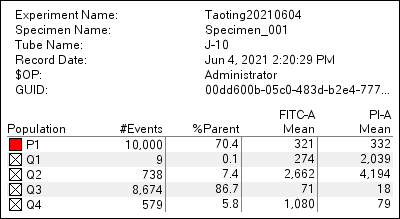

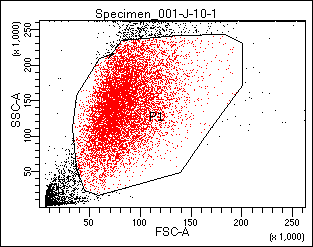

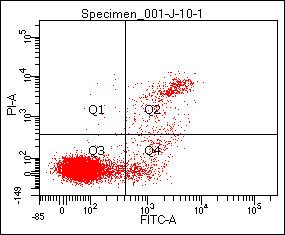

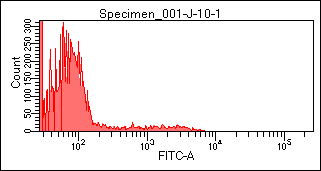

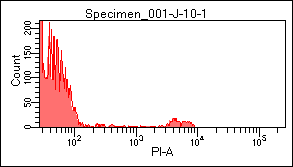

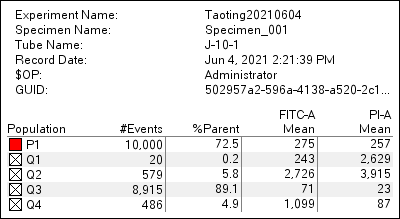

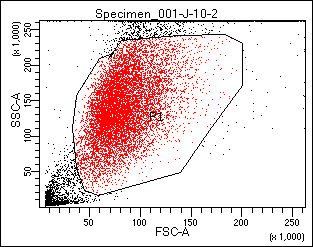

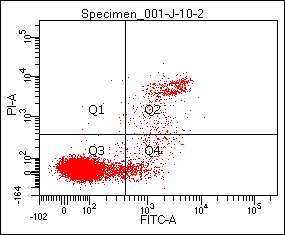

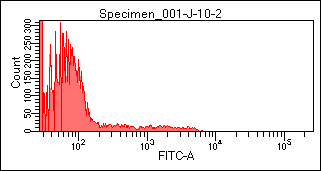

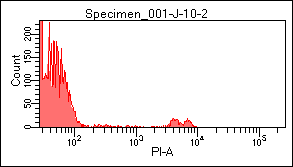

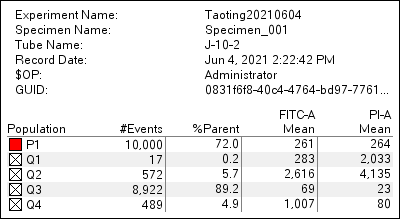

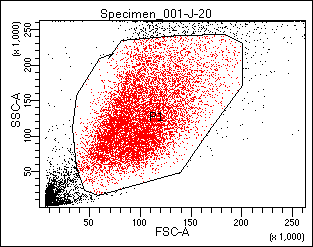

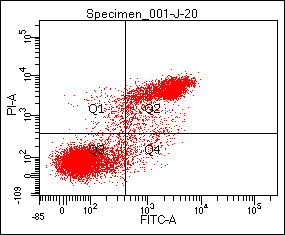

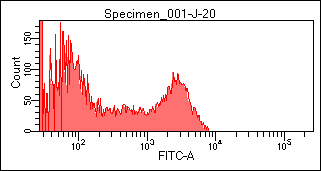

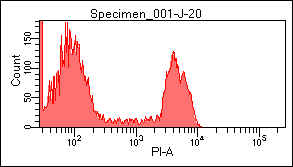

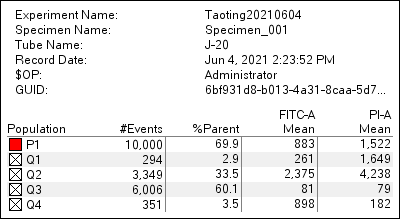

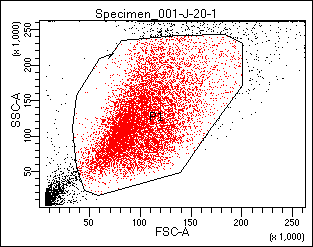

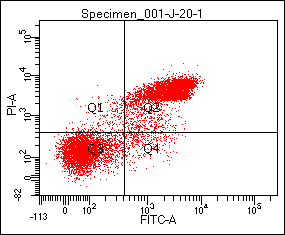

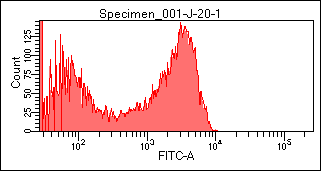

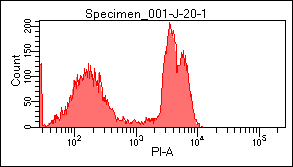

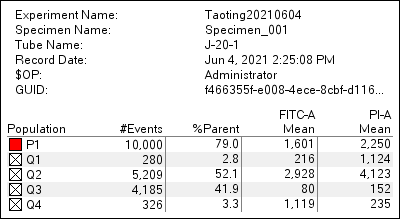

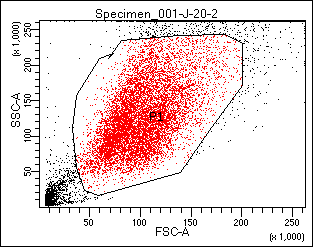

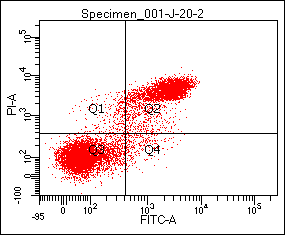

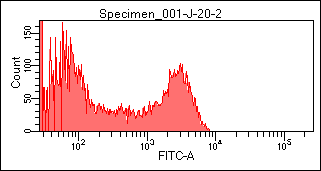

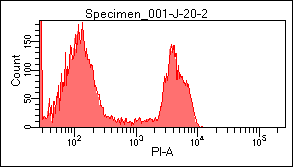

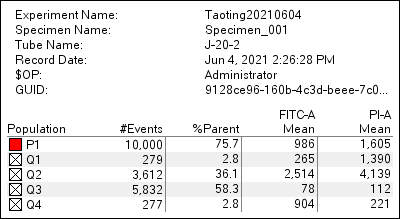

Supplement: Supplementary file 8 [file Table4.DOCX]
