# Supplementary material for: 4-Methoxydalbergione Inhibits Bladder Cancer Cell Growth via Inducing Autophagy and Inhibiting Akt/ERK Signaling Pathway
Source: Front Mol Biosci. 2022 Feb 16;8:789658. doi: 10.3389/fmolb.2021.789658 (PMC8888913; doi:10.3389/fmolb.2021.789658)
Supplement: Supplementary file 9 [file Table11.DOCX]

**Western blot description**

A：The complete band after transferring the original gel

B：The complete mark band after exposure

C: The bands after exposure to different molecular targets

D：The complete band after exposure to different molecular targets

**1-4：**UMUC3 and J82 cells were treated with 10 μM 4MOD, 5 μM SC 79 alone or combination for 72 h, and the protein levels of p-AKT, p-ERK, LC3 were detected by Western blot with GAPDH as a control.

**
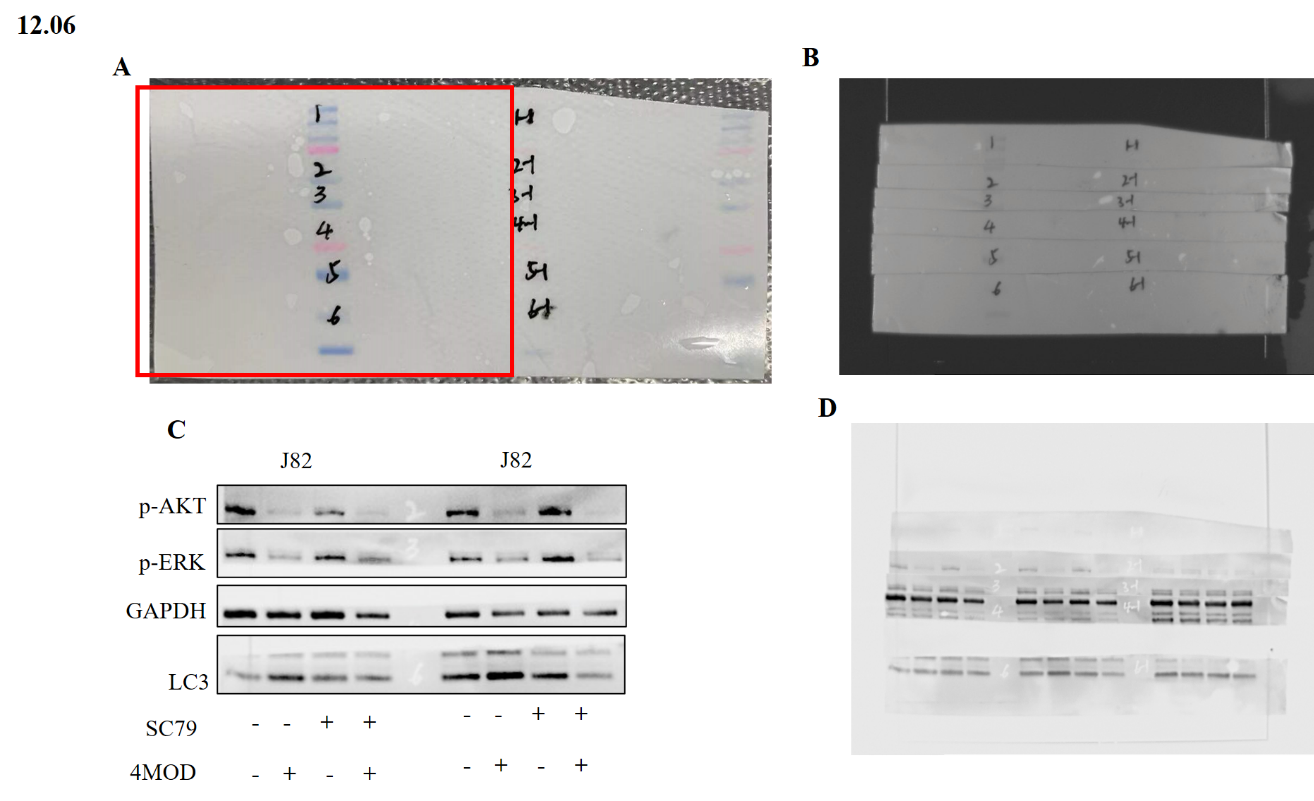
**

**2:p-AKT 3:p-ERK 4:GAPDH 5-6: LC3**

**2.**


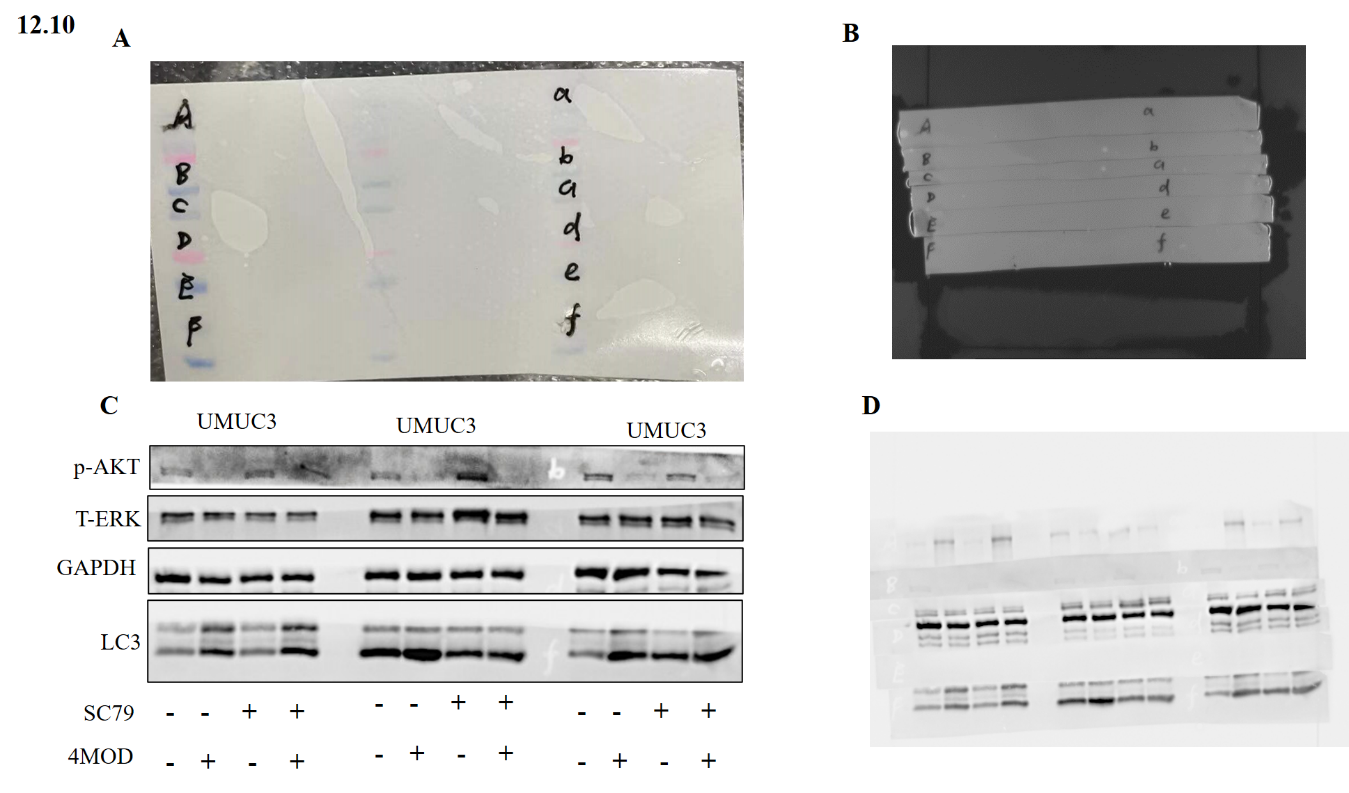


**B:p-AKT C:p-ERK D:GAPDH E-F: LC3**

**3.**


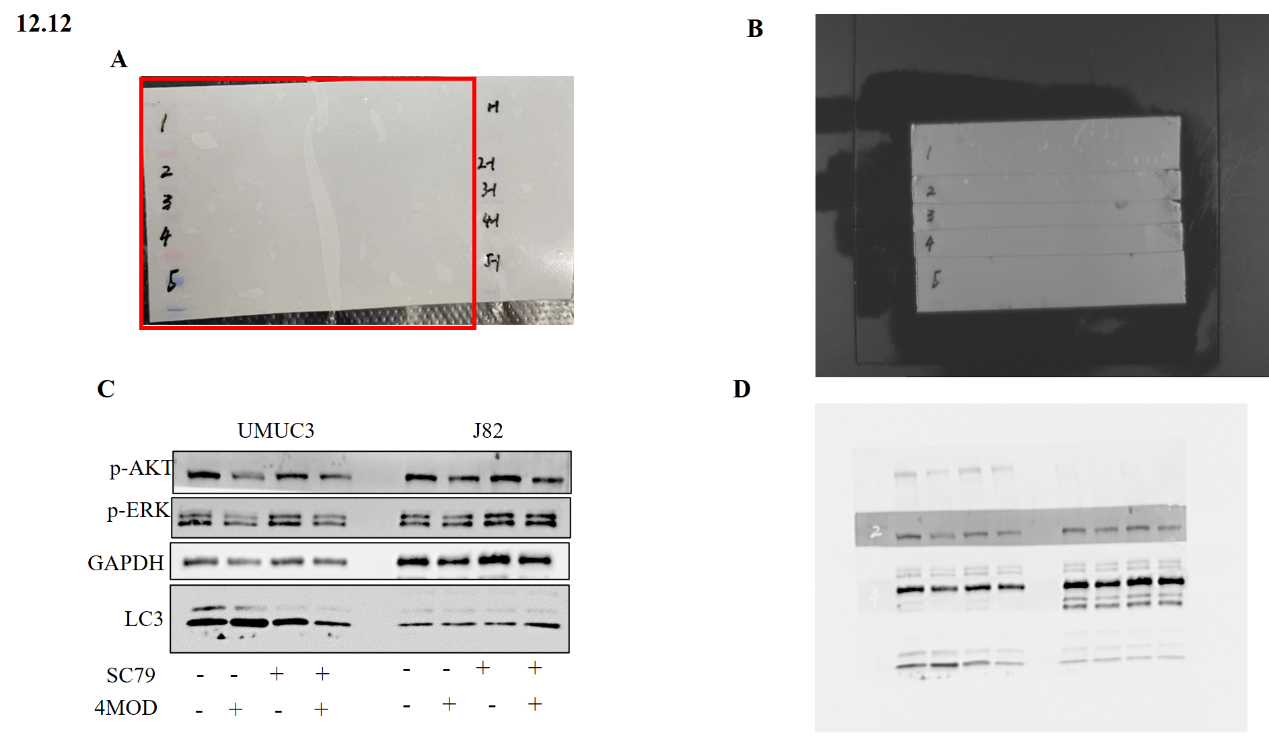


**2:p-AKT 3:p-ERK 4:GAPDH 5: LC3**

**4.**

**
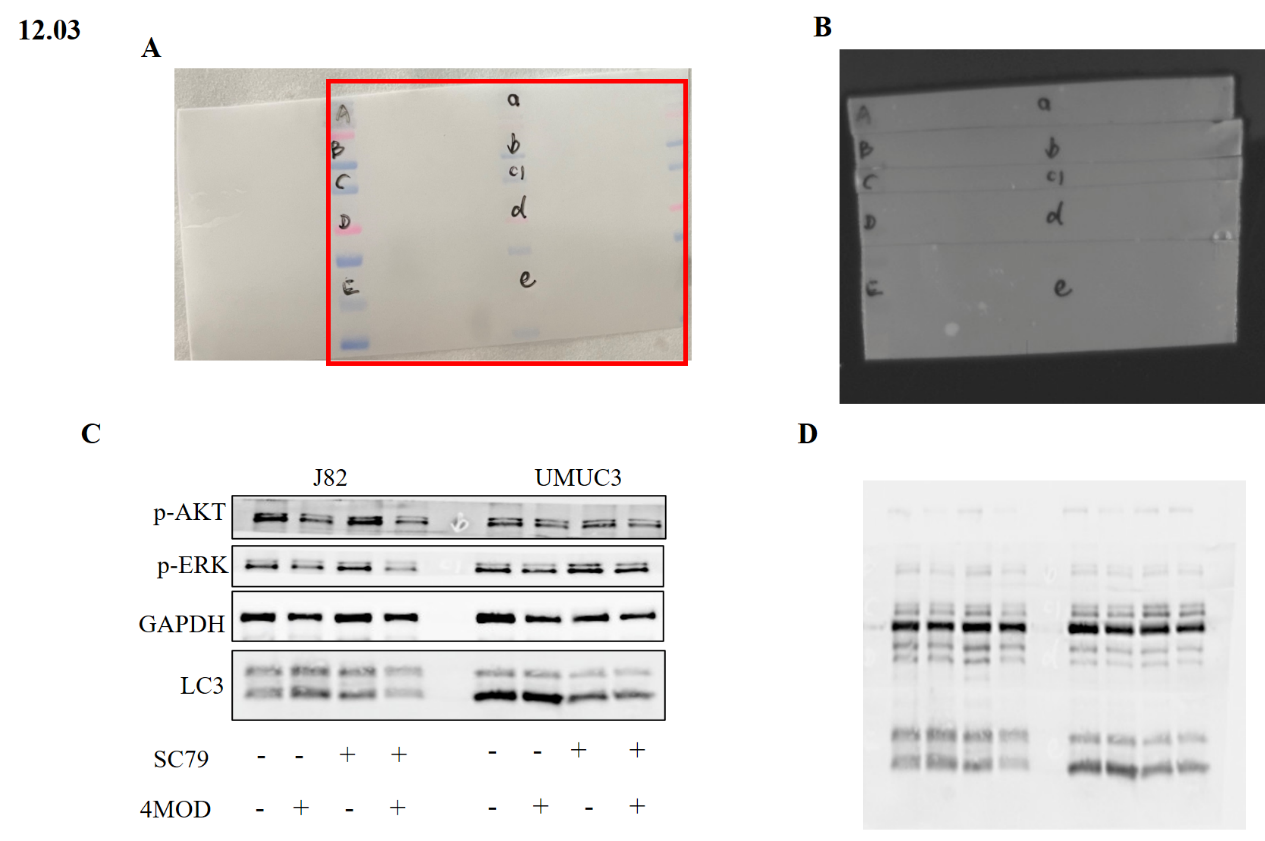
**

**B/b:p-AKT C/c1:p-ERK D/d:GAPDH E/e: LC3**

**5-7:** UMUC3 and J82 cells were treated with 10 μM 4MOD, 5 μM SC 79 alone or combination for 72 h, and the protein levels of t-AKT, t-ERK, LC3 were detected by Western blot with GAPDH as a control.

**5.**


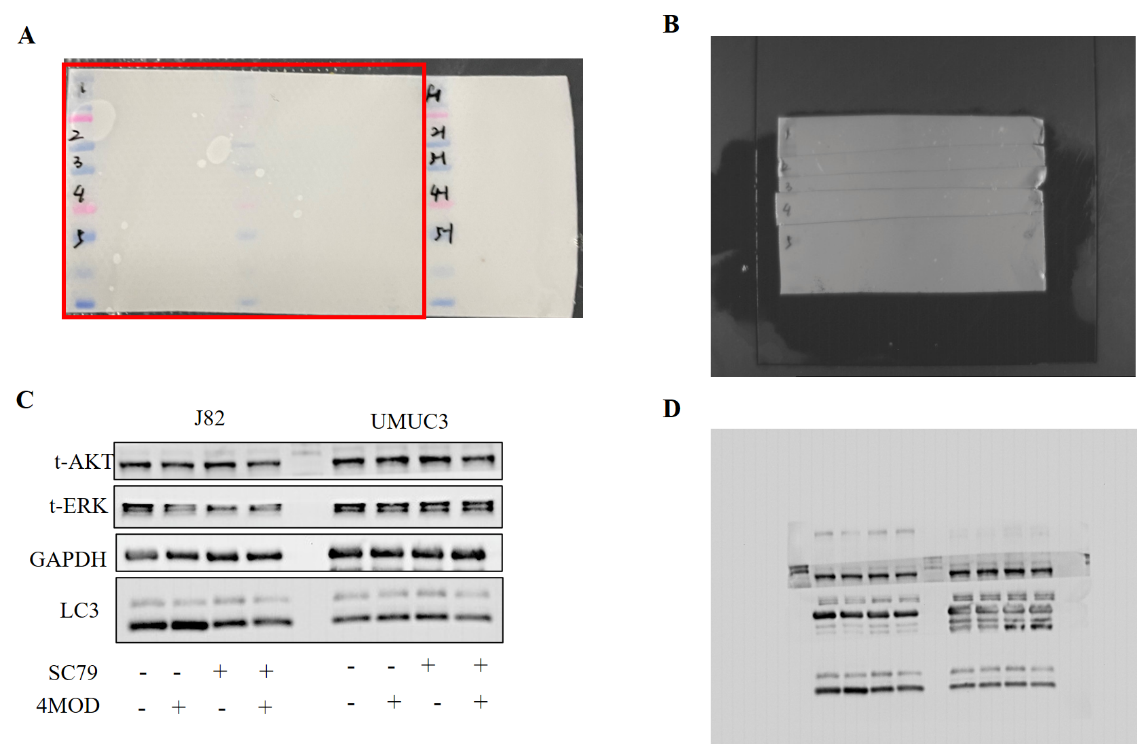


**2:p-AKT 3:p-ERK 4:GAPDH 5: LC3**

**6.**


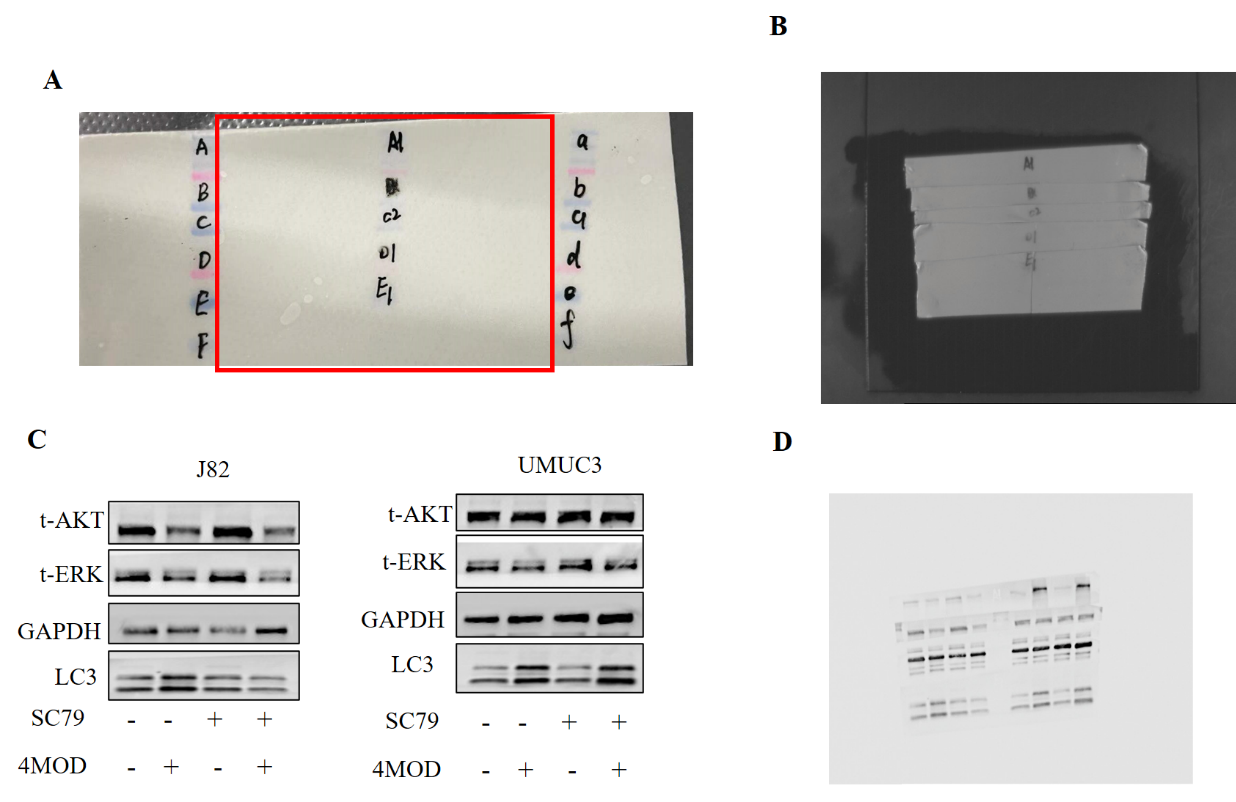


**B/B1:t-AKT C/C2:t-ERK D/D1:GAPDH E/E1: LC3**

**7.**


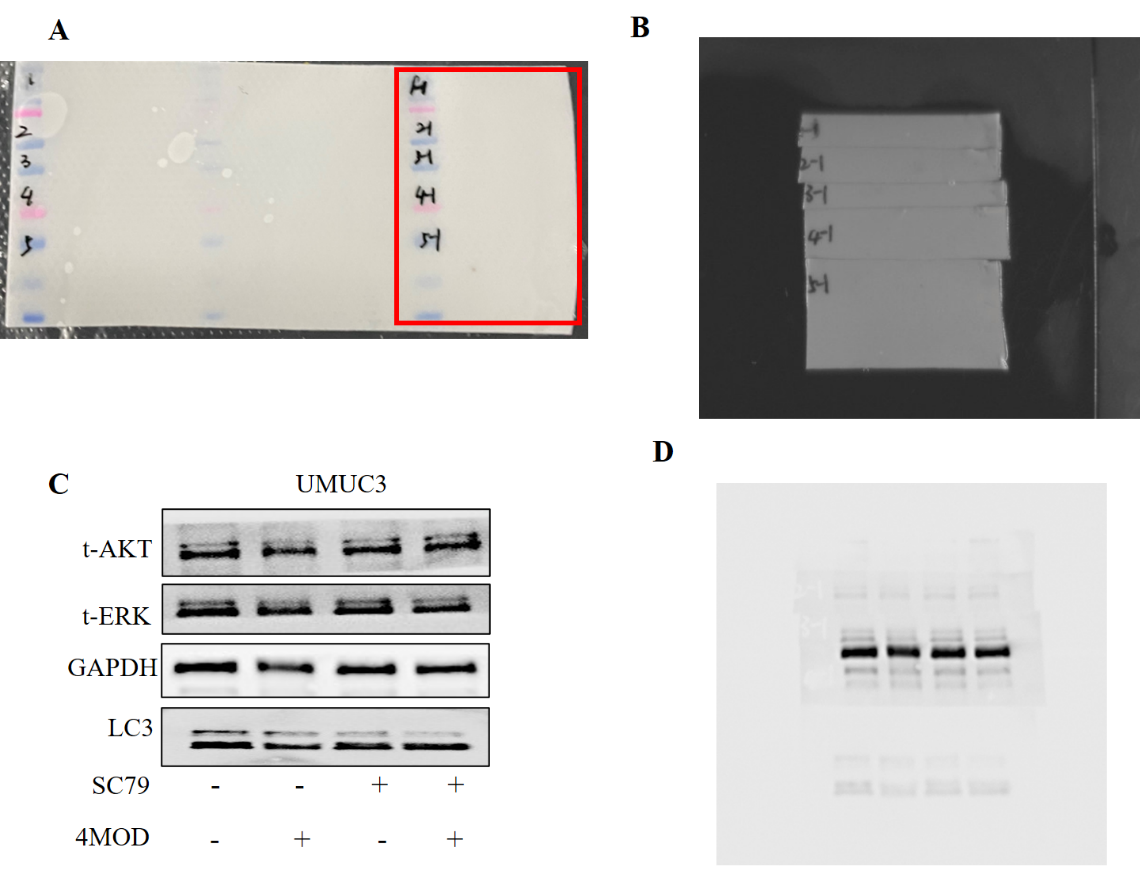


**2-1:t-AKT 3-1:t-ERK 4-1:GAPDH 5-1: LC3**

**8.**


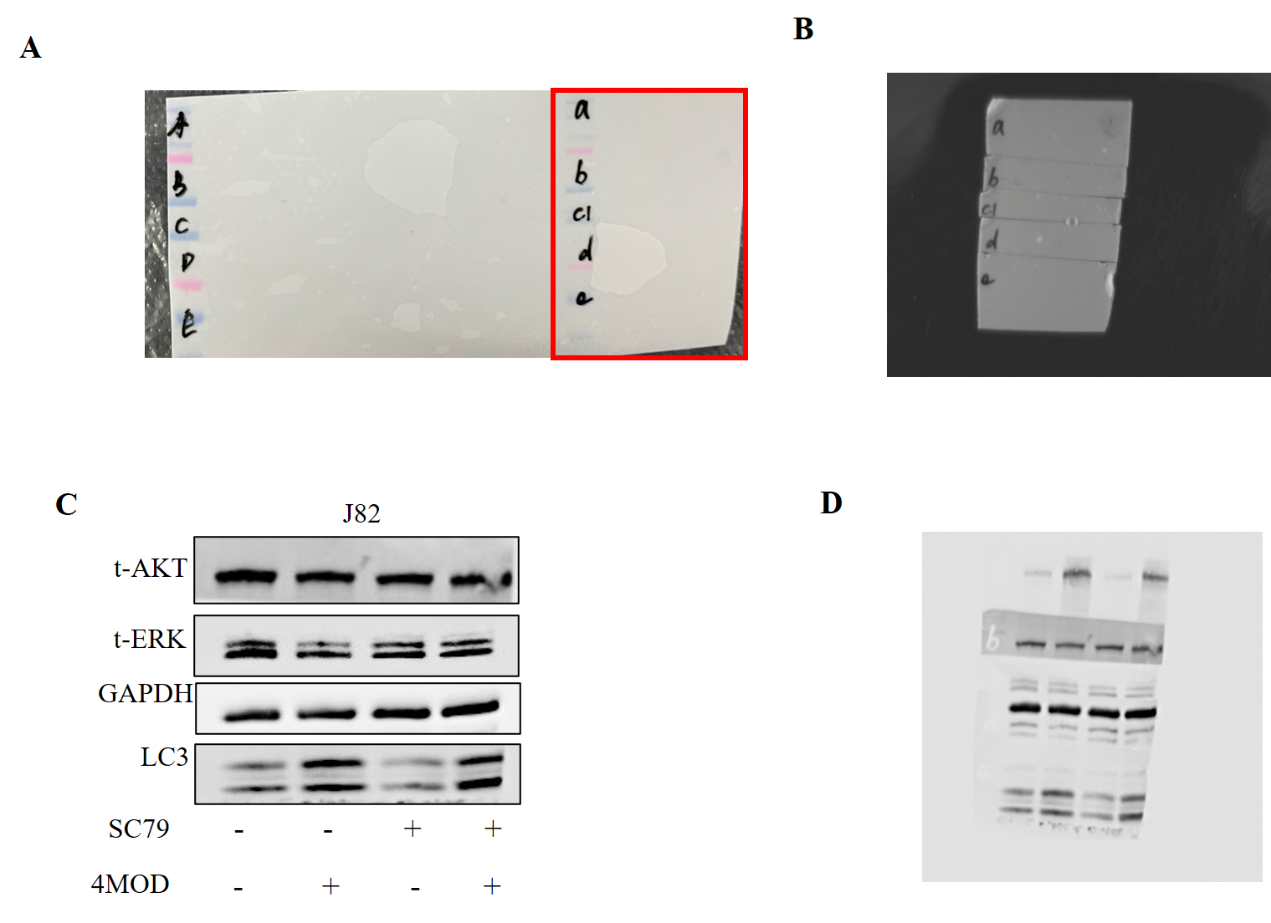


**b:t-AKT c1:t-ERK d:GAPDH e: LC3**
